# Delayed vaginal SHIV infection in VRC01 and anti-α4β7 treated rhesus macaques

**DOI:** 10.1371/journal.ppat.1007776

**Published:** 2019-05-13

**Authors:** Giulia Calenda, Ines Frank, Géraldine Arrode-Brusés, Amarendra Pegu, Keyun Wang, James Arthos, Claudia Cicala, Kenneth A. Rogers, Lisa Shirreff, Brooke Grasperge, James L. Blanchard, Stephanie Maldonado, Kevin Roberts, Agegnehu Gettie, Francois Villinger, Anthony S. Fauci, John R. Mascola, Elena Martinelli

**Affiliations:** 1 Center for Biomedical Research, Population Council, New York, New York, United States of America; 2 Vaccine Research Center, National Institute of Allergy and Infectious Diseases, Bethesda, Maryland, United States of America; 3 Laboratory of Immunoregulation, National Institute of Allergy and Infectious Diseases, National Institutes of Health, Bethesda, Maryland, United States of America; 4 New Iberia Research Center, University of Louisiana at Lafayette, New Iberia, Louisiana, United States of America; 5 Tulane National Primate Research Center, Tulane University, Covington, Louisiana, United States of America; 6 Aaron Diamond AIDS Research Center, Rockefeller University, New York, New York, United States of America; Emory University, UNITED STATES

## Abstract

VRC01 protects macaques from vaginal SHIV infection after a single high-dose challenge. Infusion of a simianized anti-α_4_β_7_ mAb (Rh-α_4_β_7_) just prior to, and during repeated vaginal exposures to SIVmac251 partially protected macaques from vaginal SIV infection and rescued CD4^+^ T cells. To investigate the impact of combining VRC01 and Rh-α_4_β_7_ on SHIV infection, 3 groups of macaques were treated with a suboptimal dosing of VRC01 alone or in combination with Rh-α_4_β_7_ or with control antibodies prior to the initiation of weekly vaginal exposures to a high dose (1000 TCID_50_) of SHIV_AD8-EO_. The combination Rh-α_4_β_7_-VRC01 significantly delayed SHIV_AD8-EO_ vaginal infection. Following infection, VRC01-Rh-α_4_β_7_-treated macaques maintained higher CD4^+^ T cell counts and exhibited lower rectal SIV-DNA loads compared to controls. Interestingly, VRC01-Rh-α_4_β_7_-treated macaques had fewer IL-17-producing cells in the blood and the gut during the acute phase of infection. Moreover, higher T cell responses to the V2-loop of the SHIV_AD8-EO_ envelope in the VRC01-Rh-α_4_β_7_ group inversely correlated with set point viremia. The combination of suboptimal amounts of VRC01 and Rh-α_4_β_7_ delayed infection, altered antiviral immune responses and minimized CD4^+^ T cell loss. Further exploration of the effect of combining bNAbs with Rh-α_4_β_7_ on SIV/HIV infection and antiviral immune responses is warranted and may lead to novel preventive and therapeutic strategies.

## Author summary

Broadly neutralizing antibodies (bNAbs) constitute a promising new strategy to prevent and/or treat HIV-1 acquisition. However, it is clear that individual bNAbs cannot be used alone as a single intervention. α_4_β_7_ blockade with a monoclonal antibody (mAb) similar to a drug approved for treatment of inflammatory bowel disease (IBD) has shown promising results against SIV infection in non-human primate studies. In the current study, we report the impact of combining the bNAb VRC01, currently in clinical testing for HIV-1 prevention, with the anti-α_4_β_7_ mAb on delaying infection of macaques with a pathogenic simian/human immunodeficiency virus (SHIV). We found that the VRC01/anti-α_4_β_7_ combination was able to significantly delay SHIV acquisition when compared with the control group, but not when compared with pretreatment with VRC01 alone. Moreover, after infection, the animals pretreated with the VRC01/anti-α_4_β_7_ combination had higher CD4 counts compared to the other treatment groups and lower amounts of virus in the gut compared to the control group. Moreover, important differences were present between the groups in the immune response to the virus in blood and mucosal tissues. More studies are needed to explore the potential synergistic effects of combining bNAbs with the α_4_β_7_ blockade for the prevention and therapy of HIV-1 infection.

## Introduction

Integrin α_4_β_7_ (α_4_β_7_) is expressed at high levels by CD4^+^ T cells trafficking to the gut associated lymphoid tissues (GALT) [1–3], a critical site for HIV-1 replication and dissemination after transmission [4–7]. α_4_β_7_^high^ CD4^+^ T cells are highly susceptible to HIV-1 infection and are preferentially depleted during acute HIV-1 and SIV infection [8–10]. Higher frequencies of α_4_β_7_^high^ CD4^+^ T cells have been correlated with increased susceptibility to HIV-1 infection in humans and SIV infection in macaques and with disease progression in both humans and macaques [11, 12]. The higher risk of HIV-1 acquisition due to prevalent HSV-2 infection has also been associated with increased levels of α_4_β_7_ expression [13–15]. Targeting α_4_β_7_ with a simianized anti-α_4_β_7_ monoclonal antibody (Rh-α_4_β_7_; mAb) prior to and during a vaginal repeated low-dose challenge (RLDC) study in rhesus macaques prevented SIV acquisition in half of the animals and delayed disease progression in those animals that did become infected [16]. Reportedly, simultaneous treatment with Rh-α_4_β_7_ and cART led to sustained viral control after cessation of all forms of therapy in at least one model of SIV infection [17]. The mechanism(s) underlying the anti-HIV-1 activity of the Rh-α_4_β_7_ mAb are poorly understood. Rh-α_4_β_7_ does not block viral entry into CD4^+^ T cells and has weak anti-HIV-1 activity *in vitro* [8, 18, 19]. We have recently shown that signaling through α_4_β_7_ can promote HIV-1 replication [20] and, in this regard, we previously demonstrated that Rh-α_4_β_7_ blocks α_4_β_7_ from adopting an active conformation that is critical for this signaling [21].

In addition, we determined that Rh-α_4_β_7_ selectively alters trafficking of CCR6^+^ CD4^+^ T cells to mucosal tissues [22] and impacts the antibody response to SIV infection when given in combination with cART [17]. Thus, interference with both immune cell trafficking and α_4_β_7_-driven viral amplification may, at least in part, explain the decrease in gut tissue SIV loads when Rh-α_4_β_7_ is administered prior to, and throughout the acute phase of infection [23].

Passive transfer of a number of broadly neutralizing antibodies (bNAbs) targeting HIV-1 envelope (Env) has been shown to protect rhesus macaques against a single high-dose inoculation with simian-human immunodeficiency virus (SHIV) [24–27] and this strategy is currently being evaluated to prevent HIV-1 acquisition in humans [28]. In particular, VRC01, a bNAb against the CD4 binding site (CD4bs) on the HIV-1 envelope [29, 30], is the first bNAb to be investigated clinically for the prevention of HIV-1 infection in adult men and women (AMP trial; NCT02716675 and NCT02568215). Moreover, VRC01 is being tested for safety in HIV-exposed infants (NCT02256631) as a potential agent to prevent mother-to-child transmission (MTCT) of HIV-1. In preclinical studies, VRC01 protected monkeys against single high-dose vaginal and rectal SHIV challenge [27] and its protective activity against repeated low-dose rectal challenges decreases after several weekly challenges [31]. In this regard, bNAb protection against repeated rectal challenges was shown to be dependent on the potency and half-life of bNAbs [31]. A mutation in the Fc domain of the antibody, which was shown to increase VRC01 half-life in both plasma and tissues, increased [32] and prolonged [31] its protective activity. Several other strategies to improve the pharmacokinetics and function of bNAbs [28] as well as the use of combinations of bNAbs or bi- and trispecific antibody-based molecules [33–35] are being tested with the ultimate goal of generating new prevention and therapeutic options against HIV-1 infection.

In the present study, we investigated the combination of VRC01 and Rh-α_4_β_7_ in a repeated vaginal challenges model using the tier 2 SHIV_AD8-EO_ [36]. This challenge virus was chosen for its multiple properties typical of pathogenic HIV-1 isolates [37], allowing us to explore the impact of the VRC01-Rh-α_4_β_7_ combination on SHIV_AD8-EO_ infection and antiviral immune responses during the acute and early chronic phase of infection. In order to detect an effect of this combination over the sterilizing protective effect of VRC01, we chose a repeated challenges model of infection and treatment with suboptimal amounts of both antibodies. The VRC01-Rh-α_4_β_7_ combination significantly delayed SHIV_AD8-EO_ acquisition, protected blood CD4^+^ T cells and altered antiviral immune responses.

## Results

### The VRC01-Rh-α_4_β_7_ combination significantly delays SHIV_AD8-EO_ vaginal infection

VRC01 has been shown to provide sterilizing protection against high-dose vaginal challenge with SHIV_SF162P3_ [27, 38]. In order to study the VRC01-Rh-α_4_β_7_ combination in a setting of suboptimal VRC01 protection we employed an inoculum 100 fold higher and half the dose of VRC01 (10 mg/kg) that resulted in delayed SHIV_AD8-EO_ acquisition for a median of 8 weeks in a repeated rectal low-dose challenge model [31]. A VRC01-alone group was used to monitor baseline VRC01 protection.

**Fig 1:**
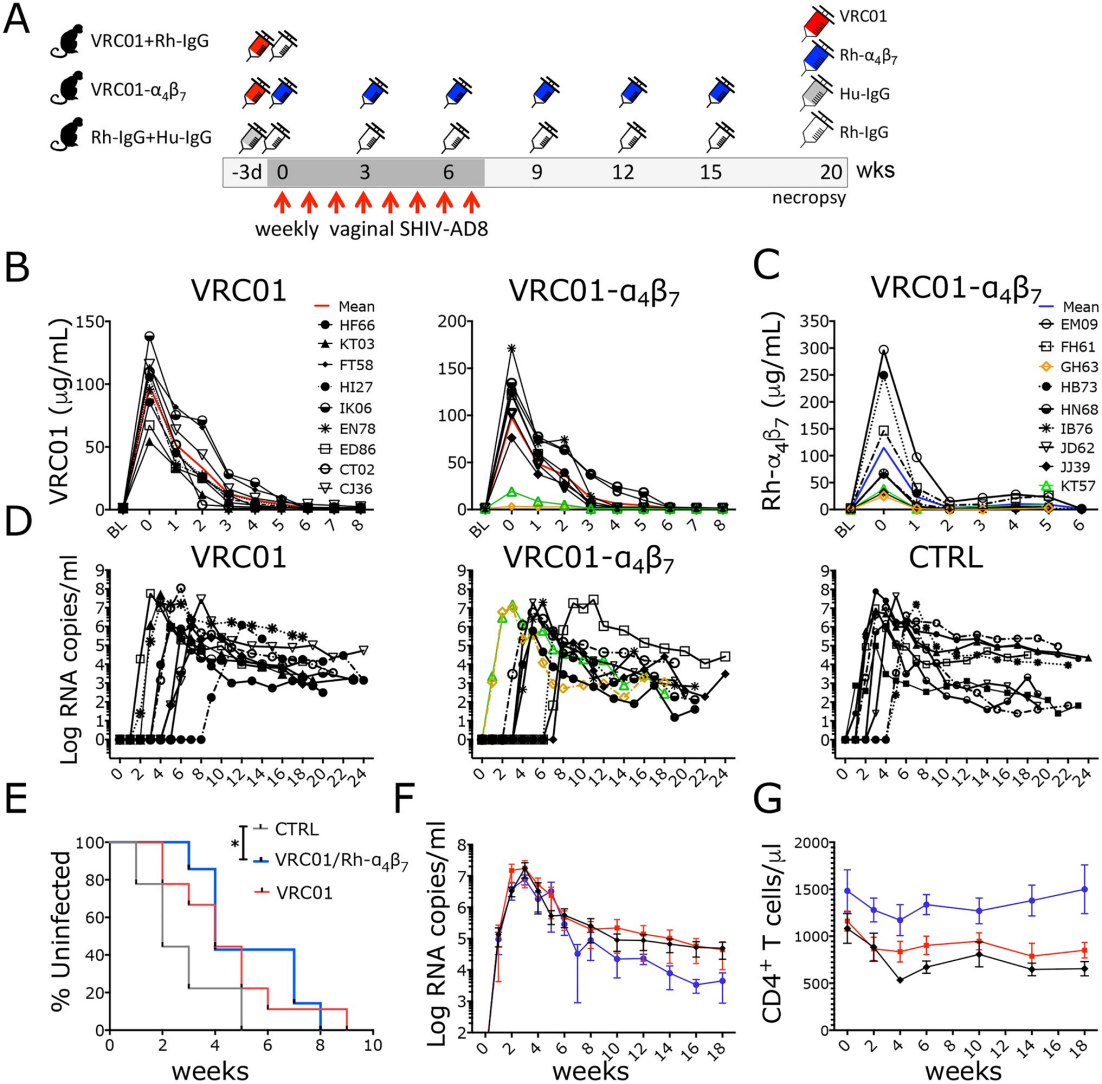
The Rh-α_4_β_7_-VRC01 combination significantly delays SHIV_AD8-EO_ acquisition. (A) Schematic of the study procedures (B-C) The concentrations of VRC01 (B) and Rh-α_4_β_7_ (C) were measured in plasma for the first 8 weeks after initiation of treatment. VRC01 levels were below the protective concentration in GH63 and KT57 (orange and green in B, respectively). BL: right before the first infusion with VRC01 and Rh-α_4_β_7_ Solid colored line (red for VRC01 and blue for Rh-α_4_β_7_) represent the mean of all macaques. (D) Log viral RNA (copies/ml) in plasma are shown from the time of the first challenge (week 0). (E) Kaplan-Meier curves generated with time to first viral detection in plasma are shown. Curves were compared with the Log-rank test (* *p*-value = 0.016; Gehan-Breslow-Wilcoxon test p = 0.015) α<0.05 (Bonferroni corrected for multiple comparisons at α<0.017 was considered significant). (F-G) Mean ± SEM of the plasma viral loads (F) and CD4^+^ T cell counts (G) are shown.

A total of 27 animals were infused with VRC01 (10 mg/kg) and Rh-α_4_β_7_ (25 mg/kg; n = 9) or with 10 mg/kg of VRC01-alone (n = 9) or with control human and rhesus IgGs (n = 9), 3 days before weekly vaginal challenges with a high-dose inoculum of SHIV_AD8-EO_ (1000 TCID_50_) until all animals became infected (Fig 1A). Rh-α_4_β_7_ infusions were repeated every 3 weeks for a total of 6 infusions. A Rh-α_4_β_7_-alone group was not included because Rh-α_4_β_7_ does not protect from high-dose challenge[39] and the levels of VRC01 rapidly decrease[27]. Thus, the impact of Rh-α_4_β_7_ on SHIV-AD8 infection can be inferred from comparison with the VRC01-only and control groups.

The peak concentrations of VRC01 on the day of the first challenge in the VRC01-only group and in the VRC01-Rh-α_4_β_7_ group were 98.31 ± 25.83 μg/ml and 97.66 ± 55.29 μg/ml (mean±SD), respectively (Fig 1B). Of note, for reasons that may be attributed to problems during the infusion, 2 macaques in the VRC01-Rh-α_4_β_7_ group had peak concentrations of VRC01 at the time of the first challenge about 10 fold lower than the mean and below the protective concentration against SHIV_SF162P3_ high-dose challenge [27, 38]. (KT57: 19.145 μg/ml and GH63: 3.350 μg/ml; respectively green and orange in Fig 1B). Thus, these 2 animals were excluded from the SHIV acquisition analysis (they acquired infection at the first challenge, as if VRC01 was absent), but not from all other post-infection analysis since VRC01 levels were undetectable in all animals by week 6 post-infection (Fig 1B). These 2 animals were not outliers in any post-infection analysis of immunological and virological parameters. Consistent with previous studies, antibodies against VRC01 (ADA) developed around week 3 after infusion in all VRC01-treated animals and levels were similar in both treatment groups (S1 Fig). The peak concentration of Rh-α_4_β_7_ in the VRC01-Rh-α_4_β_7_ group at the time of the first challenge was 112 ± 37μg/ml (mean ± SD; Fig 1C; 1 animal with peak concentrations 10 times higher than the average of the other animals was excluded from the calculation of this mean). None of the animals had plasma Rh-α_4_β_7_ concentrations below the expected range and all 9 animals were included in the analysis of the impact of the VRC01-Rh-α_4_β_7_ combination on acute and early infection parameters.

As shown in Fig 1D, all 9 animals in the control group became viremic after 1 to 5 challenges (median of 2 weekly exposures required for infection). The viral inoculum, as calculated by the infectivity in the control group, was 0.86 AID_50_.

Suboptimal dosing of VRC01 resulted in a non-significant delay in SHIV acquisition (Log-rank p = 0.074). In contrast, in the VRC01-Rh-α_4_β_7_ combination treatment, it was possible to detect a significant delay in SHIV acquisition compared to the control group (Log-rank p = 0.016; Fig 1E). However, the median number of challenges needed to infect in the two treatment groups was similar (n = 4) and the VRC01-Rh-α_4_β_7_ combination did not significantly delay acquisition compared to VRC01 alone (Log-rank p = 0.601). Of note, when the cumulative challenges to infect were compared using the Poisson exact test, none of the comparisons was statistically significant (VRC01-alone vs control p = 0.202; VRC01-Rh-α_4_β_7_ vs control p = 0.335; VRC01-alone vs VRC01-Rh-α_4_β_7_ p = 0.804). Moreover, no statistical difference was noted between the groups in the intention-to-treat analysis (S2 Fig).

FcγRs polymorphisms did not correlate with SHIV acquisition in either of the treatment groups (Fig 1 and S3 and S4 Figs lists all FcγRs polymorphisms) no effect of FcγRs polymorphisms was noted in any other post-infection analysis. Peak plasma viral load (VL) did not significantly differ either between the treatment groups or when comparing the treatment groups with the control group (Fig 1F and S5 Fig). Set-point VL was ~1 Log_10_ lower in the VRC01-Rh-α_4_β_7_ group compared with the other 2 groups (Fig 1F). However, the difference did not reach statistical significance (p = 0.072 at week 18 p.i.). Confirming previous reports on the effects of Rh-α_4_β_7_ [23, 40], peripheral CD4^+^ T cells were protected and CD4 counts were significantly higher in the VRC01-Rh-α_4_β_7_ when compared with the other 2 groups (treatment adjusted for time; 2-way Anova p = 0.005; Fig 1G). This is at least partially due to Rh-α_4_β_7_-driven lymphocytosis, as we demonstrated in naïve macaques [22], and explains the higher baseline CD4 count of the VRC01-Rh-α_4_β_7_ group.

**Fig 2:**
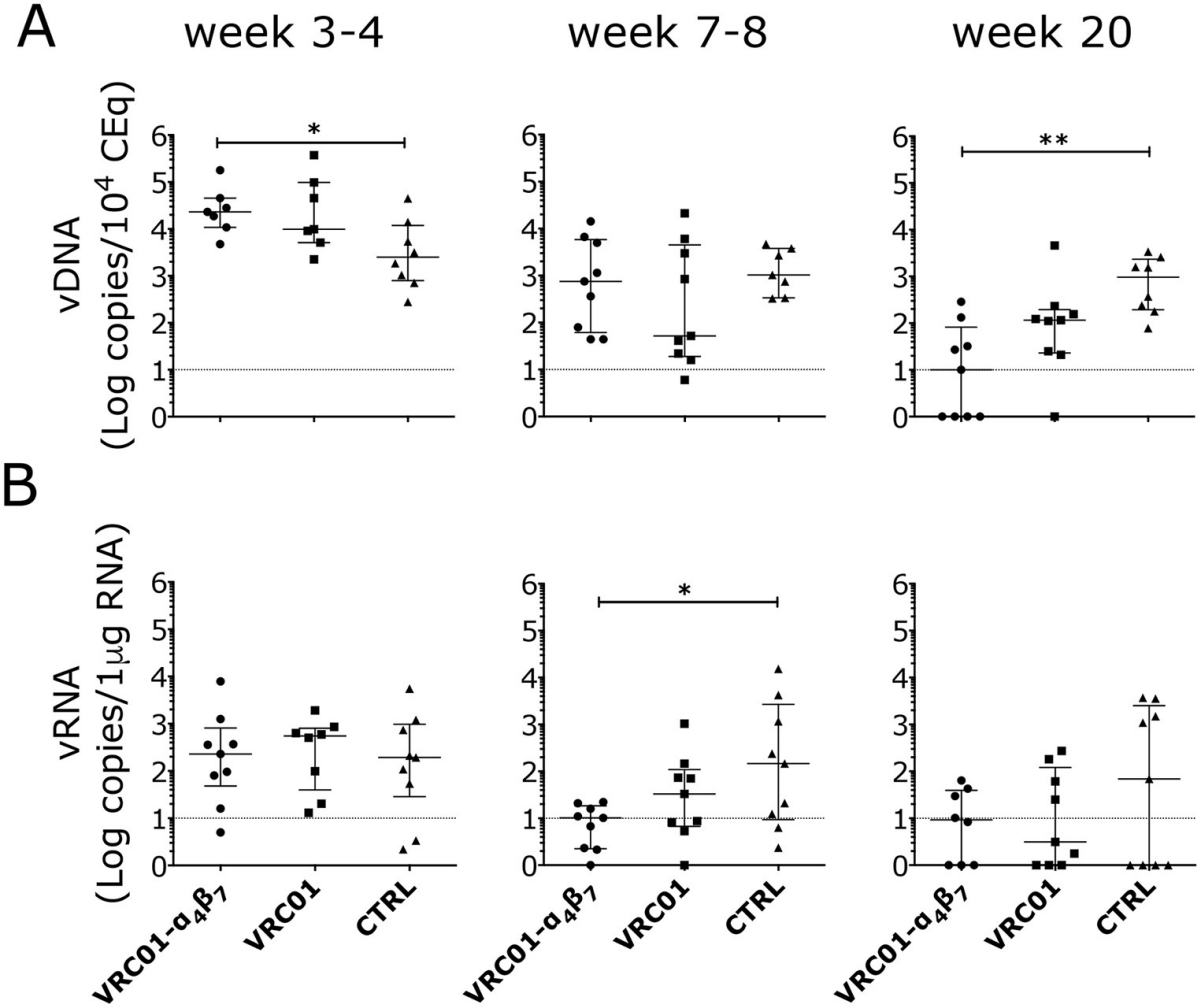
The Rh-α_4_β_7_-VRC01 combination reduces viral DNA and RNA in the gut. Copies of SIV DNA (A) and RNA (B) from colorectal biopsies at the indicated times after infection were quantified by *gag*-qPCR (normalized on albumin content) and by RT-qPCR (normalized on RNA content) respectively. Bars represent median ± IQR. The dotted line indicates the lower limit of detection (LLOD) of the assay. Data from the treatment groups were compared with the control by Kruskal-Wallis test and the results of the Dunn’s multiple comparisons post-hoc test are shown (*p*-value of * α<0.05 and ** α <0.01 were considered significant).

Although gut SIV DNA loads were slightly higher in both treatment groups compared with controls at week 3–4 p.i. (2–3 weeks after 1^st^ virus detection in plasma), by week 20 p.i., VRC01-Rh-α_4_β_7_ treated animals had more than 10-fold lower amounts of cell-associated SIV DNA in the gastrointestinal (GI) tract compared to the controls (Fig 2A). SIV RNA loads in the GI tract of the VRC01-Rh-α_4_β_7_ treated animals were also 100 times lower, on average, than in the control group during the post-acute phase (week 7–8 p.i.; Fig 2B). However, by week 20 p.i. several animals in the control group had undetectable SIV-RNA levels in the GI tract and the difference with the VRC01-Rh-α_4_β_7_ lost its significance. Interestingly, no significant differences in SIV loads were found by directly comparing the VRC01-Rh-α_4_β_7_ combination group and the VRC01-alone group and the SIV loads in the VRC01-only group often averaged between the other 2 groups. Vaginal SIV DNA and RNA loads did not differ in the acute nor in the early chronic phase of the infection among the treatment groups (S6 Fig). Finally, although the SIV-DNA loads at necropsy (around 20 weeks p.i.) were on average slightly lower in the jejunum and iliac lymph nodes of the VRC01-Rh-α_4_β_7_ group compared with the other 2 groups, the differences were not significant (S7 Fig). No differences were detected in other tissues and lymph nodes at necropsy (S7 Fig).

### VRC01-Rh-α_4_β_7_ decreases IL-17-producing cells in acute infection and increases IFN-γ-producing cells in the chronic phase

Mononuclear cells isolated from blood and colorectal biopsies in the acute phase of infection (week 3–4 p.i.) and from ileum, jejunum and colorectal tissue at necropsy (chronic phase) were stimulated with PMA/ionomycin to determine the frequency of IL-17 (acute and chronic phase samples) and IFN-γ, IL-2, TNFα and IL-21-producing cell subsets (chronic phase samples). T lymphocytes and NK cells were analyzed since they express high levels of α_4_β_7_ and thus, they may be directly impacted by Rh-α_4_β_7_ treatment [22]. Moreover, bNAbs’ activity may be driven, at least in part, by NK-mediated ADCC [41].

**Fig 3:**
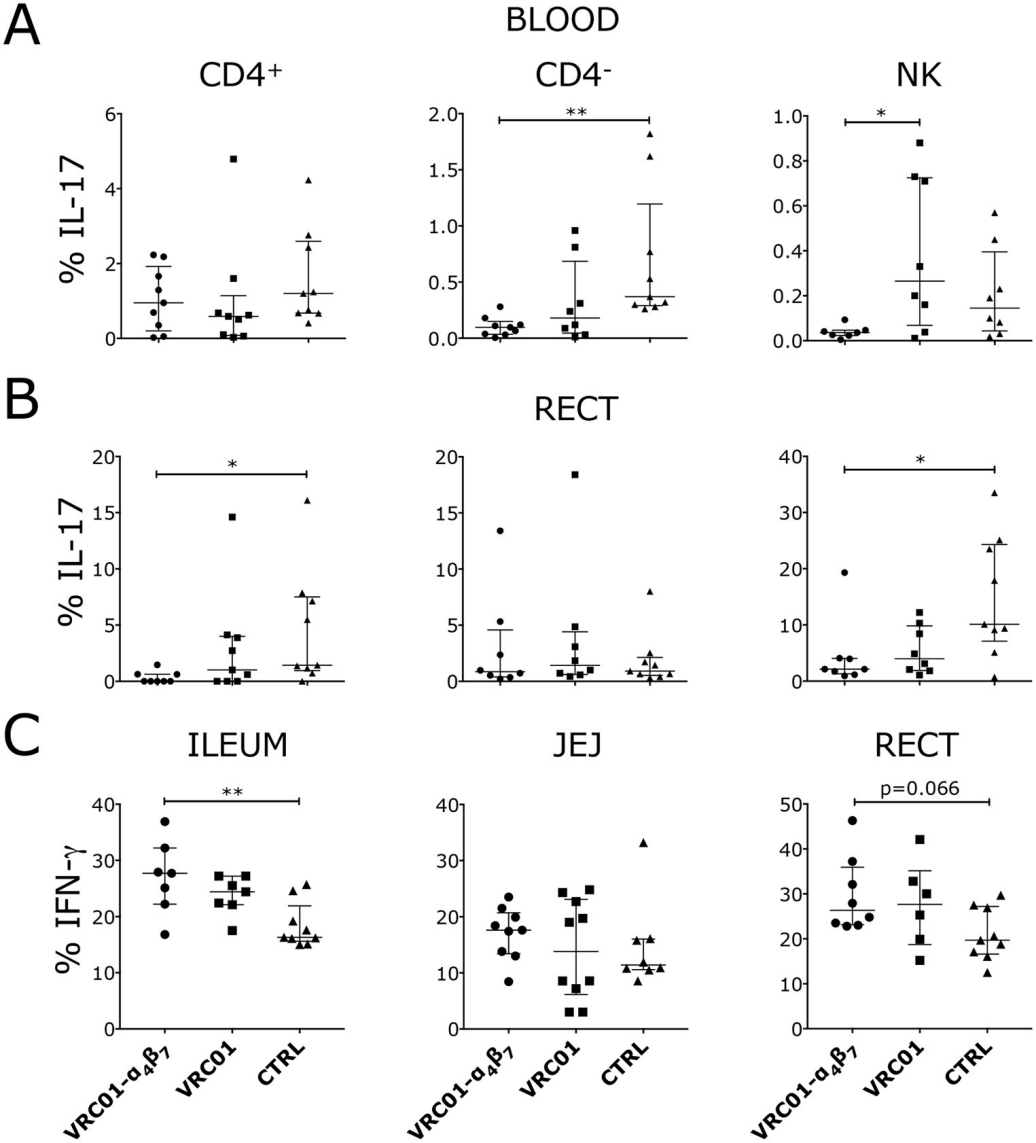
Decreased IL-17 and increased IFN-γ producing cells in the gut of Rh-α_4_β_7_-VRC01-treated macaques. (A-B) The frequency of IL-17-secreting cells within the indicated subsets in blood (A) and colorectal biopsies (B) collected 2 weeks after the first detection of virus in plasma (3–4 weeks post-infection) are shown. (C) The frequency of IFN-γ-secreting cells within CD8^+^ T cells in the indicated tissues at necropsy (~22–24 weeks p.i.) are shown. (A-C) NK-like cells were defined as CD3^-^NKG2A^+^ in the blood and CD3^-^NKp44^+^ in the colorectal tissue. Bars represent median ± IQR. Data from the treatment groups were compared with the control by Kruskal-Wallis test and the results of the Dunn’s multiple comparisons post-hoc test and the Mann-Whitney test to compare the treatment groups between each other are shown (*p*-value of * α<0.05 and ** α<0.01 were considered significant).

VRC01-Rh-α_4_β_7_ treated animals had significantly lower frequencies of IL-17 producing CD4^-^ T cells in blood and of IL-17 producing CD4^+^ T cells and CD3^-^ NKp44^+^ NK cells in the colorectal tissue compared to the control group (Fig 3A and 3B; gating in S8 Fig and baseline frequencies in S9 Fig). For these subsets, the difference did not reach significance when compared with the VRC01-only group. However, the frequency of IL-17-producing NKG2A^+^ NK cells in blood was significantly lower in the VRC01-Rh-α_4_β_7_ group compared with the VRC01-only group (Fig 3A, S8 and S9 Figs). In contrast, in the chronic phase, the frequency of IFN-γ-producing CD8^+^ T cells was higher in the VRC01-Rh-α_4_β_7_ group compared with the control group in the small intestine (Fig 3C; gating in S8 Fig and baseline values in S9 Fig). No significant differences in IFN-γ-producing cells were noted in blood or upon direct comparison of the VRC01-only group with the VRC01-Rh-α_4_β_7_ group nor between the VRC01-only group and the control. No significant differences in IL-17, IL-2, TNFα and IL-21-producing cells were noted in these tissues in the chronic phase of infection.

**Fig 4:**
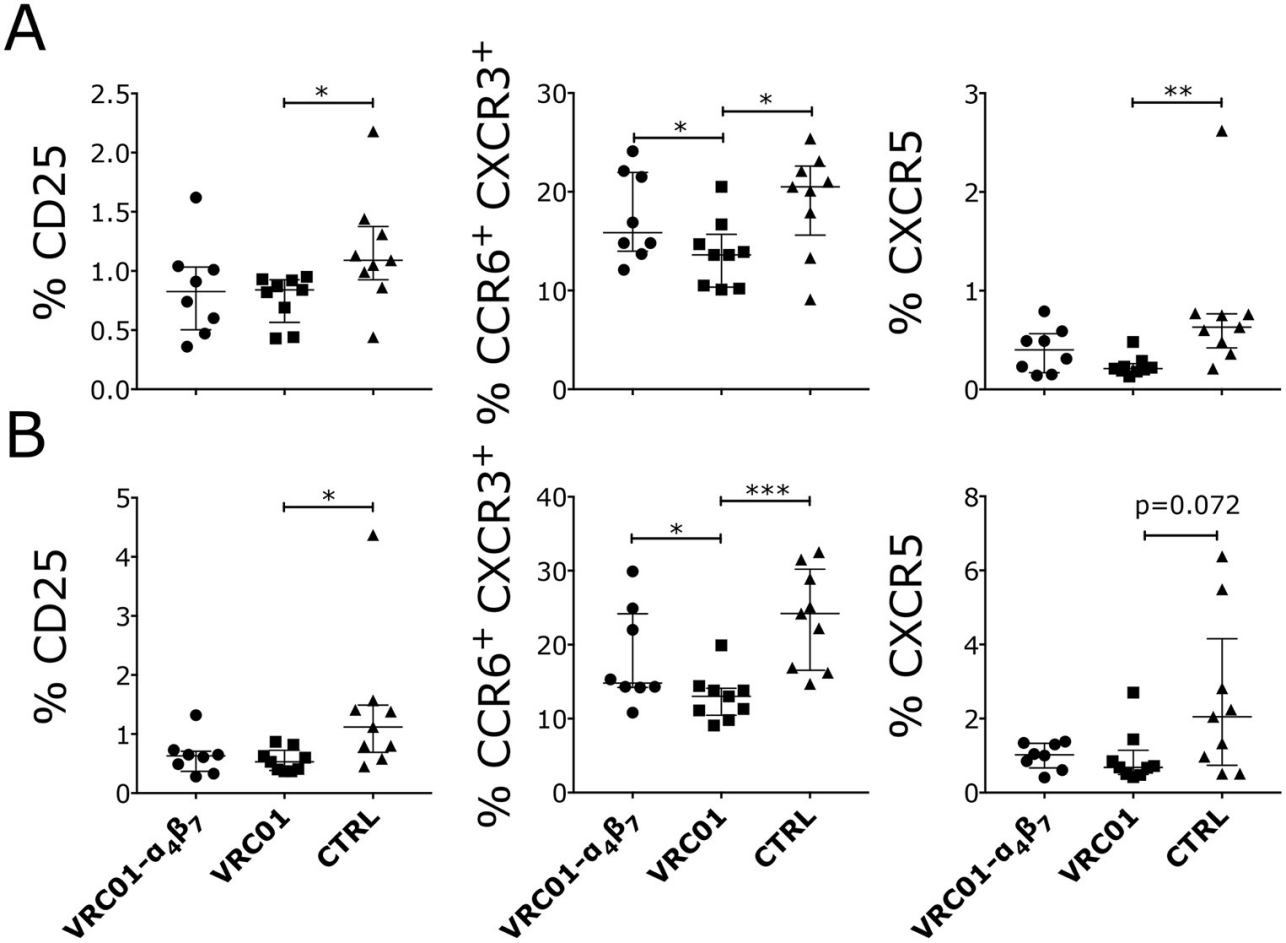
Decreased frequencies of CD25^+^, CCR6^+^ CXCR3^+^ and CXCR5^+^ T cells in the lymph nodes of VRC01-pretreated, SHIV-infected macaques. (A-B) At necropsy, cells were isolated from inguinal lymph nodes and analyzed by flow cytometry. The frequencies of subsets within the CD4^+^ (A) and CD4^-^ (B) T cells that were significantly different in any of the treatment groups compared to the controls by Kruskal-Wallis test are shown. Cells from 1 animal (HB73) in the VRC01-Rh-α_4_β_7_ group were lost during data acquisition. The results of the Dunn’s multiple comparisons post-hoc test and the Mann-Whitney test to compare the treatment groups between each other are shown (*p*-value of * α<0.05, ** α<0.01 and *** α<0.001 were considered significant). Bars represent median ± IQR.

Interestingly, when we investigated how the treatments had impacted immune cells in the lymph nodes at necropsy, we found that the VRC01-only group had a lower frequency of CD25^+^ T cells (both CD4^+^ and CD4^-^, Fig 4) compared with the control group. Rh-α_4_β_7_ in the VRC01-Rh-α_4_β_7_ may have interfered with this decrease since the difference was not significant between the VRC01-Rh-α_4_β_7_ group and the controls. Moreover, we found that the VRC01-treated animals had lower frequencies of CXCR3/CCR6 double positive CD4^-^ and CD4^+^ T cells compared with both the VRC01-Rh-α_4_β_7_ and the control groups (Fig 4A and 4B). Finally, the VRC01-only treated animals had lower CXCR5^+^ CD4^+^ follicular T cells in the lymph nodes compared with the controls (Fig 4A). No other differences were noted among CD103^+^, CD69^+^, and Treg-like CD127^-^CD25^+^ cells within the CD4^+^ or CD4^-^ cell subsets in the lymph nodes between the groups. Phenotyping of mononuclear cells isolated from blood during the chronic phase of infection showed that Rh-α_4_β_7_ treatment was associated with an increase of CCR6^+^/CD95^-^ CD4^-^ cells compared to both control groups (S10 Fig).

### T cell responses in the VRC01-Rh-α_4_β_7_ are primarily directed against the V2-loop of the SHIV_AD8-EO_ envelope protein

Blood T cell responses were analyzed against pooled 15-mer overlapping peptides from the consensus B envelope and Gag proteins around week 18 post-infection. Interestingly, T cell responses against the envelope peptides in the VRC01-Rh-α_4_β_7_ group were virtually undetectable and significantly lower than the T cell responses in the control group (S11 Fig). T cell responses in the VRC01 group were more similar to the control group, but the difference between the VRC01-only and VRC01-Rh-α_4_β_7_ groups reached statistical significance only for TNFα and IL-22-secreating CD4^+^ T cells (S11A Fig). The responses to Gag peptides were generally lower than those to the envelope peptides at this stage of infection in all groups and differences between groups could not be determined.

**Fig 5:**
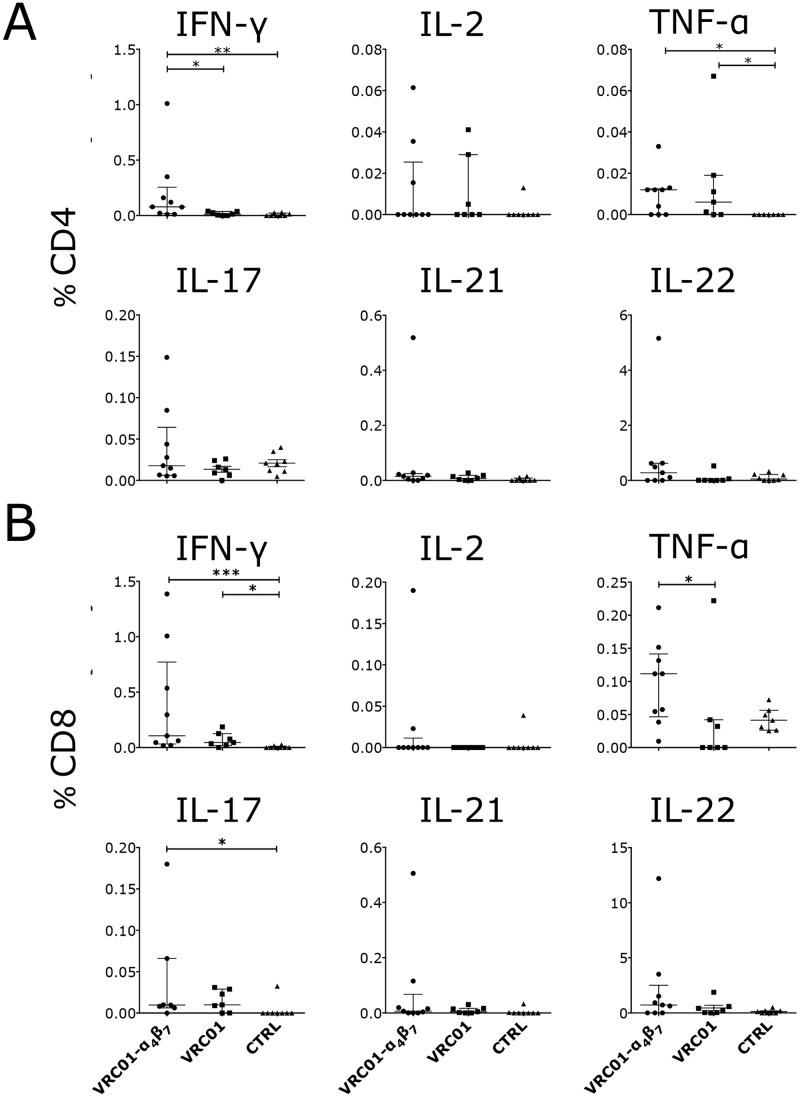
T cell responses to the SHIV_AD8-EO_ V2-loop peptides are higher in Rh-α_4_β_7_-VRC01 treated macaques. A-B) PBMCs isolated around 18 weeks post infection were stimulated with a pool of 7 20mers with 14aa overlap peptides spanning the V2 loop of the SHIV_AD8-EO_ envelope for 5 hours. The frequency of cells secreting the indicated cytokines are shown for the CD4^+^ (A) and CD8^+^ (B) T cell subsets after subtraction of the baseline values (in absence of peptides). The results of the Dunn’s multiple comparisons post-hoc test (after the Kruskal-Wallis test controlled for multiple comparisons) and the Mann-Whitney test to compare the treatment groups between each other are shown (*p*-value of * α<0.05, ** α<0.01 and *** α<0.001 were considered significant). Bars represent median ± IQR. Note: ROUT (Q = 1%) analysis for outliers excludes the highest % of CD4^+^ and CD8^+^ T cells producing TNF-α in the VRC01 group. In the cleaned data set, the differences between the VRC01-only group and the control group in the CD4^+^ T cell subset is not significant and the difference between the VRC01-α_4_β_7_ group and the VRC01 group in the CD8^+^ T cell subset becomes highly significant. There are no other changes due to outliers analysis.

We previously reported that the V2 loop of gp120 mediates high affinity binding to α_4_β_7_ [42, 43]. Since the sequence of the consensus B envelope differs substantially from the sequence of the SHIV_AD8-EO_ envelope in the V1V2 region (S12 Fig) and a specific response against the V2-loop was found in the Rh-α_4_β_7_-treated animals of the cART-Rh-α_4_β_7_ study [17], we synthetized seven 20-mer, 14aa overlapping peptides spanning the V1V2 region that differs between the SHIV_AD8-EO_ and the consensus B envelope and used them to probe T cells and antibody responses. Interestingly, the VRC01-Rh-α_4_β_7_ macaques had higher frequencies of IFN-γ-producing CD4^+^ T cells in response to the V1V2 peptide pool than both the VRC01 and control groups (Fig 5A). In contrast, TNFα-producing CD4^+^ T cells and IFN-γ-producing CD8^+^ T cells were higher in both treatment groups compared with the control group (Fig 5A and 5B). A higher IL-17 response in the CD8^+^ T cell subset was also noted in the VRC01-Rh-α_4_β_7_ group compared to the controls (Fig 5B). Finally, a higher frequency of TNF-α-CD8^+^ T cells was present in the VRC01-Rh-α_4_β_7_ group compared with the VRC01-only group. In summary, higher T cell responses were detected against the V1V2 loop in the VRC01-Rh-α_4_β_7_ group particularly compared to control animals. Interestingly, the frequency of CD8^+^ T cells secreting TNF-α in response to the V1V2 peptides inversely correlated with viral load (S13 Fig), suggesting a possible role played by V1V2 responses in controlling viral replication in the VRC01-Rh-α_4_β_7_ group.

Total anti-HIV envelope antibodies were tested against the HIV-Bal envelope protein and no differences were noted between the groups. Moreover, a peptide scan against consensus B envelope peptides (with the 8 peptides corresponding to the V1V2 loop replaced by the 7 SHIV-AD8-specific peptides that we had synthetized) was carried out on sera from 4 animals in each group with the highest antibody responses. No clear differences in the response to specific regions of the envelope were noted between the groups (S14 Fig).

## Discussion

bNAbs are being tested in the clinic for the prevention and therapy of HIV-1 infection. VRC01 is the first to reach efficacy testing and other bNAbs will soon follow [44, 45]. However, it is clear that individual bNAbs cannot be used alone as a single intervention. Combinations of more bNAbs or bi- or tri-specific molecules need to be employed to achieve better and more durable protection from HIV-1 acquisition [33, 46–48]. Moreover, recent data suggest that bNAbs treatment may impact immune responses to infection [49–51]. This feature represents a potential new therapeutic approach toward an HIV-1 cure. Rh-α_4_β_7_ has also demonstrated the ability to partially prevent SIV infection in macaques [16] and treatment of SIV infected macaques with Rh-α_4_β_7_ in combination with cART has shown its potential utility in inducing long-term control of SIV replication without eradicating the virus [17]. Nonetheless, much more needs to be understood about the ability of bNAbs and α_4_β_7_-blockage to impact immune responses against SIV/HIV.

The present study represents the first investigation of the combination of a bNAb and Rh-α_4_β_7_. We aimed to determine how this dual-treatment might alter key features of acute and early-chronic infection, including antiviral immune responses. In order to observe such effects beyond the powerful protective activity of VRC01, we performed the study in a setting of suboptimal amounts of VRC01 against repeated challenges with a relatively high viral inoculum (compared to previous studies [31]) of a pathogenic SHIV. By design, we challenged the animals until all acquired infection with the dual aim in mind of determining the impact on SHIV acquisition and investigate the immunomodulatory effect of the antibodies on acute infection. Unfortunately, the lack of an Rh-α_4_β_7_-alone group precluded a thorough assessment of the effects of Rh-α_4_β_7_ on susceptibility and acute infection parameters to compare with previous studies in other macaque models of HIV[16, 40]. Moreover, although very low to undetectable concentrations of VRC01 were present during the acute phase of the infection, it is difficult to ascertain the contribution of Rh-α_4_β_7_ to the post-infection differences noted between the treatment groups. Finally, the exclusion of 2 macaques due to technical issues with VRC01 infusion from the acquisition analysis, but not from the post-infection analysis is a limitation of the study. It was justified given that all the animals had very low plasma VRC01 levels at the post-infection time points analyzed and none were outliers. Nonetheless, their exclusion from all the analysis may have been more correct.

Overall, more studies are needed to determine if and how Rh-α_4_β_7_ may increase the protective activity of suboptimal dosing of bNAbs. The protective effect of the VRC01-Rh-α_4_β_7_ combination was not significantly higher than the VRC01-alone group. However, excluding the two animals in the VRC01-Rh-α_4_β_7_ group that exhibited extremely low levels of plasma VRC01, the VRC01-Rh-α_4_β_7_ combination was able to significantly delay infection in our model in contrast to the VRC01 alone treatment group. Since a decrease in CCR6^+^ CD4^+^ T cells in mucosal tissues is one of the major effects of the Rh-α_4_β_7_ in naïve macaques [22], the small effect that the Rh-α_4_β_7_ had on SHIV_AD8OE_ acquisition may be explained, in part, by a decreased availability of this important cell target (CCR6^+^ CD4^+^ T cells) at the mucosal portal of entry. A similar mechanism may have been at play in the protection shown by the Rh-α_4_β_7_ against LDC with SIVmac251 in a previous study [16]. The smaller protective effect of the Rh-α_4_β_7_ may be due to the substantially higher inoculum used in our study compared to Byrareddy et al [16]. The inoculum we used corresponded to 0.86 AID_50_ compared to less than 0.2 AID_50_ used in Byrareddy et al [16], and it was ~10 fold higher based on in vitro TCID_50_ (determined concurrently in our laboratory on both stocks) and ~300 fold higher based on p27 content. The higher inoculum may have lowered the transmission bottleneck, decreasing the relative importance of target cell availability for infection. Larger studies with a more physiologically relevant, low-dose viral challenge model will be needed to precisely quantify the incremental effect of combining Rh-α_4_β_7_ and VRC01 over VRC01 on SHIV acquisition.

Interestingly, we were able to determine that some of the effects of the Rh-α_4_β_7_ that were previously described [16, 23, 40] are retained by the VRC01-Rh-α_4_β_7_ combination in our SHIV model. They include the ability to protect circulating CD4^+^ T cells, a modest effect on the viral set-point and a decrease in the gut viral load. However, we did not find a decrease viral load in any other tissue or lymph nodes as it was described for the Rh-α_4_β_7_-alone in the SIV model [23]. Protection of the CD4 counts may be due to a small, non-significant effect on chronic plasma viremia with the VRC01-Rh-α_4_β_7_ combination (Fig 1F), in addition to the reduced gut viremia and the lymphocytosis effect of the Rh-α_4_β_7_ previously reported [22].

Interestingly, most of the differences that we noted are significant only when the VRC01-Rh-α_4_β_7_ group is compared with the control group and the data from the VRC01-only group fell in between. This suggests that the presence of minimal quantities of VRC01 at the time of infection may have increased the impact of Rh-α_4_β_7_ on virologic and immune parameters, hinting at the possibility of a synergistic effect of the two antibodies. Nonetheless, the lack of Rh-α_4_β_7_-alone group precludes the systematic evaluation of a synergistic activity and this, also, will require further investigation.

Of note, we found that during the acute phase of infection (which coincided with the Rh-α_4_β_7_-treatment) the VRC01-Rh-α_4_β_7_ group had a lower frequency of IL-17 producing T cells in the gut. This is not surprising since most Th17 are α_4_β_7_^+^ [9] and may be due to the effect of the Rh-α_4_β_7_ on CCR6^+^ T cells that we noted in absence of infection [22]. This early decrease in IL-17 may contribute to lower immune activation during the acute phase of infection, since higher IL-17 during acute infection has been associated with AIDS progression [52]. A reduced inflammatory state in mucosal tissues may help explain the apparent protection of IFN-γ producing cells in the large and small intestine during chronic infection in the VRC01-Rh-α_4_β_7_ group.

Moreover, reduced trafficking of lymphocytes to the gut-associated lymphoid tissue (GALT) may lead to a decrease in lymphoid aggregates (as recently described in [53]) reducing overall immune cell priming. This may help explain the overall decrease in SIV-specific T cell responses that was observed in the VRC01-Rh-α_4_β_7_ group when peptide pools for the entire envelope and Gag proteins were used.

Perhaps because the Rh-α_4_β_7_ was administered during the earliest stages of the infection, we did not see an impact on the anti-gp120 antibody responses as was described in the ART-Rh-α_4_β_7_ combination study [17]. However, treatment with the VRC01-Rh-α_4_β_7_ combination increased T cell responses against the V2 region of the envelope and our data suggest a role for these responses in maintaining virologic control. How and why this happens requires further investigation. We speculate that Rh-α_4_β_7_ changes the immunogenicity of the V2-loop region of the envelope, perhaps by interfering with common mechanisms of gp120 processing and MHC-II presentation in antigen presenting cells. This may be due to the high expression of integrin α_4_β_7_ on activated DCs and macrophages [54, 55].

The increase in IFN-γ producing cells in the gut suggests a protective activity of the VRC01-Rh-α_4_β_7_ combination on the gut immune system and helps explain the long-term survival of SIV infected animals treated with Rh-α_4_β_7_ during acute infection (reported at CROI 2018 by J. Arthos [56]). Boosting effective mucosal immune responses may also help explain the virologic control seen when the Rh-α_4_β_7_ was used in combination with cART as therapeutic approach in Byrareddy et. al, Science 2016 [17]. This control was not replicated in other similar studies (as reported by Di Mascio and Fauci at AIDS2018 [57]). Nonetheless, our study adds to the considerable amount of work that supports the immunomodulatory effect of Rh-α_4_β_7_.

Interestingly, the only VRC01-specific effect we observed appeared in the lymph nodes, where in the VRC01-only treated animals, we detected significantly lower frequencies of CD25^+^ and CXCR5^+^ CD4^+^ T cells. In the VRC01-Rh-α_4_β_7_ group, this effect was still present, but less pronounced. This further supports the published observations that suggest a direct impact of bNAbs on the antiviral immune response [50, 58]. Even so, the amount of time the animals were exposed to meaningful concentrations of VRC01 during infection was very short and the data should be interpreted with caution. Data from breakthrough infections in the AMP study will help shed light on the effects of low levels of VRC01 during acute HIV-1 infection.

In conclusion, a suboptimal dose of VRC01 in combination with the Rh-α_4_β_7_ significantly delayed infection and impacted the availability and distribution of immune cell subsets as well as the T cell responses to the virus. VRC01 and Rh-α_4_β_7_ impact HIV-1 infection by distinct mechanisms, neither of which is fully understood. This study provides the first insight into the combination of these antibodies and contributes to our understanding their effect on HIV-1 infection. Future studies should address how other bNAbs combinations with Rh-α_4_β_7_ could be harnessed in different therapeutic and preventive settings to fight HIV-1 infection.

## Materials and methods

### Ethics statement

A total of 27 adult female Indian rhesus macaques (Mamu A*01, B*08 and B*17 negative; average weight 8.1 kg, range: 4.5, 13.05 and age 9.5 years, range: 3.9, 18) were socially housed (2 animals/cage), indoors in climate-controlled conditions with a 12/12-light/dark cycle until the time of the first challenge. After initiation of the viral challenges, all animals were single housed to avoid cross-infections. All of the macaques in the study were previously assigned to the SPF breeding colony for varying lengths of time. Parity was equal among the groups. Animals were monitored twice daily to ensure their welfare. Any abnormalities, including those of appetite, stool, behavior, were recorded and reported to a veterinarian. The animals were fed commercially prepared monkey chow twice daily. Supplemental foods were provided in the form of fruit, vegetables, and foraging treats as part of the TNPRC environmental enrichment program. Water was available at all times through an automatic watering system. The TNPRC environmental enrichment program is reviewed and approved by the IACUC semiannually. Veterinarians at the TNPRC Division of Veterinary Medicine have established procedures to minimize pain and distress through several means. Monkeys were anesthetized with ketamine-HCl (10 mg/kg) or tiletamine/zolazepam (6 mg/kg) prior to all procedures. Preemptive and post procedural analgesia (buprenorphine 0.01 mg/kg) was required for procedures that would likely cause more than momentary pain or distress in humans undergoing the same procedures. The above listed anesthetics and analgesics were used to minimize pain or distress associated with this study in accordance with the recommendations of the Weatherall Report. The animals were euthanized at the end of the study using methods consistent with recommendations of the American Veterinary Medical Association (AVMA) Panel on euthanasia and per the recommendations of the IACUC. Specifically, the animals were anesthetized with tiletamine/zolazepam (8 mg/kg IM) and given buprenorphine (0.01 mg/kg IM) followed by an overdose of pentobarbital sodium. Death was confirmed by auscultation of the heart and pupillary dilation. None of the animals became severely ill or died prior to the experimental endpoint. The TNPRC policy for early euthanasia/humane endpoint was included in the protocol in case those circumstances arose. All studies were approved by the Animal Care and Use Committee of the TNPRC (OLAW assurance #A4499-01; protocol P0180-3639) and in compliance with the Animal Welfare Act and the Guide for the Care and Use of Laboratory Animals. TNPRC is accredited by the Association for Assessment and Accreditation of Laboratory Animal Care (AAALAC#000594).

### Macaque treatments

Macaques were divided in three groups of 9 animals each. Animals were administered intravenously 1) 1 injection of 10 mg/kg of VRC01 + 1 injection of 25 mg/kg of Rh-α_4_β_7_ mAb; followed by 5 additional injections of 25 mg/kg of Rh-α_4_β_7_ mAb every 3 weeks 2) 1 injection of 10 mg/kg of VRC01; 3) 1 injection of 10mg/kg of control Human IgG and 25 mg/kg of control rhesus IgG. Starting 3 days after treatment, macaques were challenged weekly with intravaginal administration of SHIV_AD8-OE_ (1,000 TCID_50_/challenge) for 8 weeks. Blood was collected weekly to monitor infection as described below. After two consecutive positive SIV PCRs (3–4 weeks post infection; acute phase) fresh rectal biopsies and blood were used for cell isolation as described below. Subsequently, blood was collected every two weeks throughout the study. Rectal and vaginal samples were also collected at 7–8 weeks post infection (post-acute phase). At necropsy, blood, lymph nodes, gut, brain, vaginal and cervical tissues were harvested and used for cell isolation.

### Measurement of plasma levels of VRC01 and Rh-α_4_β_7_

Levels of VRC01 were measured as described in [27]. Levels of rhesus Rh-α_4_β_7_ antibody in macaque plasma were measured using the α_4_β_7_-expressing human T cell line HuT-78 (NIH AIDS Reagent Program, Division of AIDS, NIAID, NIH: HuT 78 from Dr. Robert Gallo) in a flow cytometry-based assay as described in [16, 22] using the standard curve method. Briefly, HuT 78 cells were first incubated for 2–3 days in complete RPMI 1640 media containing 100 nM retinoic acid to increase the surface expression of α_4_β_7_. Cells (150,000/condition) were stained with LIVE/DEAD Aqua dye (Thermo Fisher Scientific, Waltham, MA) for live/dead discrimination, incubated for 30min at 4°C with the plasma to be tested (1:10 diluted in PBS) obtained from macaques from the VRC01-α_4_β_7_ treatment group before (baseline, BL) and up to 6 weeks after treatment. Cells were then washed and incubated for 30 min at 4°C with anti-rhesus IgG1 (NHP Resource Center, antibody 7H11, in house biotinylated with EZ-link NHS-biotin (Thermo Fisher Scientific) following the manufacturer’s instructions), washed again and resuspended in neutravidin-PE (Thermo Fisher Scientific) for 20 min at 4°C. PE fluorescence was analyzed on a flow cytometer. For the standard curve, baseline plasma was pooled and spiked with serial dilutions of Rh-α_4_β_7_ (2,500 μg/ml– 0 μg/ml).

### Anti-VRC01 and anti-Rh-α_4_β_7_ antibodies

Levels of anti-VRC01 antibodies were measured as described in [32]. Levels of anti-Rh-α_4_β_7_ antibodies were measured via lamda light chain detection assay. ELISA plates were coated with Rh-α_4_β_7_ (10μg/ml) overnight at 4°C, washed and blocked with TBS 2% BSA 0.1% Tween20 for 2 hours at room temperature. Test plasma was serially diluted in dilution buffer (starting at 1:10 then serial 1:4 dilutions), 100 μl applied to the plates and incubated 1 hour at room temperature. Plates were washed and incubated with anti-Ig human lambda light chain-biotin (Miltenyi), which does not recognize the kappa chain of the Rh-α_4_β_7_ 1 hour at room temperature. Plates were washed and incubated with diluted streptavidin-HRP (Invitrogen) 1 hour at room temperature. Plates were washed and enzymatic activity detected by adding TMB substrate and read on a luminometer at 450nm. Endpoint was the highest dilution with OD 2-fold higher the pre-treatment sample.

### SHIV_AD8-EO_ stock generation and titration

The full-length proviral plasmid pSHIVAD8-EO was a gift from Dr. Malcom Martin. Virus stocks were prepared by transfecting 293T cells with 5μg of the pSHIV_AD8-EO_ molecular clone using Lipofectamine 2,000 (Invitrogen). Culture supernatant was collected 60 hours later, clarified by centrifugation (300g 10 minutes, 4°C) and used to infect CD8-depleted, PHA-activated rhesus macaque PBMC. Cells were incubated overnight in 293T supernatants, washed and resuspended in RPMI 10% FBS medium for 10 days. Supernatants from parallel cultures were pooled on day 7, clarified by centrifugation (10,000g, 15 minutes, 4°C), aliquoted, and stored at -80 °C. The resulting stock was titrated in PHA-activated rhesus macaque PBMC.

### SIV viral loads

Macaque infection was confirmed by SIVgag nested PCR on PBMC as described [49]. Plasma samples were obtained from EDTA-treated whole blood and used for the determination of plasma VL by SIVgag qRT-PCR [50] (quantitative Molecular Diagnostics Core, AIDS and Cancer Virus Program Frederick National Laboratory). DNA and RNA were extracted from snap frozen tissues using DNeasy/RNeasy blood and tissue kits (Qiagen) following the manufacturer’s instructions. Tissue viral DNA loads were quantified using the standard curve method and normalized by albumin copy numbers by Gag-qPCR as described in [59]. For tissue RNA loads, 1μg of total RNA was retrotranscribed to DNA using the VILO Kit (Thermo Fisher) quantified by Gag-qPCR [59].

### Cell isolation and flow cytometry

3–4 weeks post infection, PBMCs were isolated using Ficoll-Hypaque density gradient centrifugation and cells from rectal biopsies were isolated by enzymatic digestion in HBSS containing 2mg/mL Collagenase IV (Worthington Biochemical) and 1mg/ml of Human Serum Albumin (Sigma-Aldrich), shaking at 37°C for 50 minutes. The resulting cell suspension was passed through a 40μm cell strainer and washed with PBS. PBMCs and rectal cells were then stimulated with 60ng/ml PMA, 0.5μg/ml Ionomycin and 5μg/ml Brefeldin A (BFA) for 4 hours at 37°C, stained with LIVE/DEAD Aqua viability dye (Thermo Fisher Scientific) and incubated with a cocktail of different panels of monoclonal antibodies as listed in the tables in S15 Fig.

At the time of necropsy, PBMCs were isolated by Ficoll-Hypaque density gradient centrifugation. Enzymatic digestion was used to isolate cells from jejunum, ileum, colorectal, vaginal and cervical tissues as described above. Spleen and LNs (axillary, mesenteric, inguinal, iliac) were cut in small pieces and passed directly through a 40μm cell strainer. Isolated cells were washed, frozen for phenotyping and stimulation experiments at a later time. For PBMC stimulation experiments, up to 3x10^6^ cells/sample were thawed, plated on a plate pre-coated with 2.5μg/ml goat anti mouse (GAM) IgGs and cross linked with 10μg/ml anti-CD28 and anti-CD49d antibodies (Sigma Aldrich). Cells were stimulated either with SIVMAC239 GAG peptide pool (1μg/ml; 125 15mers with 11 aa overlap, AIDS Reagents Program, Division of AIDS, NIAID, NIH), HIV-1 Consensus B Env Peptide Set (2μg/ml; 211 15mers with 11 aa overlap, AIDS reagents program, Division of AIDS, NIAID, NIH) or a SHIV_AD8OE_ specific Env V1-V2 peptide pool (2μg/condition; 7 20mers with 14 aa overlap, Peptide 2.0, Chantilly, VA). Pooled cells stimulated with 1μg/ml PMA/Ionomycin were used a positive activation control. One hour later 10μg/ml BFA and monensin (GolgiStop, BD Biosciences) were added to each well. After 5 hours, cells were transferred to a FACS plate and stained with the panels listed in S15 Fig. To maximize CD107a detection, antibody staining was performed during stimulation. For gut tissue stimulation experiments, cells isolated from colorectum, jejenum and ileum where thawed and stimulated with 60ng/ml PMA, 0.5μg/ml Ionomycin and 5 μg/ml Brefeldin A (BFA) for 4 hours at 37°C, stained with LIVE/DEAD Aqua viability dye (Thermo Fisher Scientific) and incubated with a cocktail of different panels of monoclonal antibodies as listed in the tables in S15 Fig. For phenotyping experiments, cells were thawed and stained with the panels listed in tables in S15 Fig.

### Pepscan

Peptide scan was performed against consensus B envelope peptides with the 8 peptides corresponding to the V1-V2 loop replaced by the 7 SHIV_AD8OE_-specific peptides that we had synthetized was performed on serum samples from the 4 animals from each group showing the highest antibody response by ABL Inc.

### Statistics

To analyze the differences in SHIV acquisition, the survival curves generated with time to first viral detection in plasma from each treatment group were compared to each other directly and each with the curve from the control group using the Log-rank (Mantel-Cox) test and with the Gehan-Breslow-Wilcoxon test with p value Bonferroni corrected for multiple comparisons. The cumulative number of challenges needed to infect in each group was also compared to the cumulative number in each other group by Poisson exact test. In the Log rank, Gehan-Breslow-Wilcoxon and Poisson exact tests, data from KT57 and GH63 were excluded to allow a fair comparison of infection acquisition between the 2 treatment groups. A survival curve showing intention-to-treat analysis including these 2 animals is shown in S2 Fig. Viral loads and CD4 counts were compared by two-way ANOVA for repeated measures. To address whether either treatment had a significantly different effect on infection and immunological parameters than the control group, data was analyzed using Kruskal-Wallis non-parametric test adjusted for multiple comparisons followed by the Dunn’s multiple comparisons post-hoc test. To address whether treatment groups differed from each other a Mann-Whitney unpaired t-test was performed. All analyses were performed using the GraphPad Prism software V7. Significant *p*-values of α<0.05 (*), α<0.01 (**) and α <0.001 (***) are indicated.

## Supporting information

S1 Fig Anti-drug antibodies levels. Mean ± SEM of the endpoint titers of anti-VRC01 antibodies in the VRC01-alone and VRC01-α_4_β_7_ group are shown at baseline and for the first 8 weeks past-infusion. B) Endpoint titers of anti-Rh-α_4_β_7_ antibodies are shown for the VRC01-α_4_β_7_ group from baseline to the necropsy.
(PDF)

S2 Fig Intention-to-treat acquisition analysis. Kaplan-Meier curves generated with time to first viral detection in plasma are shown. Curves were compared with the Log-rank test and Gehan-Breslow-Wilcoxon test and no comparison was significant after Bonferroni correction for multiple comparisons.
(PDF)

S3 Fig CD16 and CD64 polymorphisms in the macaques. RNA was isolated from PBMC of each animal and cDNA prepared. Gene-specific PCRs were run, and the product sequenced. Animals are listed in order of treatment with the first 9 animals belonging to the VRC01 + Rh-α_4_β_7_ group, then the 9 animals from the VRC01-only group and finally the 9 animals in the control group. The alleles in yellow had 2 different nucleotides present at more than a single SNP. They were inferred based on the allele frequency in the population. In bold are the 2 animals with very low VRC01 concentrations.
(PDF)

S4 Fig CD32a genotype of the macaques. RNA was isolated from PBMC of each animal and cDNA prepared. Gene-specific PCRs were run and the product sequenced. Animals are listed in order of treatment with the first 9 animals belonging to the VRC01 + Rh-α_4_β_7_ group, then the 9 animals from the VRC01-only group and finally the 9 animals in the control group. In green are highlighted the animals with the most common allotype. In bold are the 2 animals with very low VRC01 concentrations.
(PDF)

S5 Fig No difference in peak plasma viral load among the treatment groups. Highest level of SIV RNA copies in plasma reached within the first 5 weeks of infection in each animal is shown. Bars represent median ± IQR.
(PDF)

S6 Fig No difference in vaginal tissue viral load among the treatment groups. Copies of SIV DNA/ 10^4^ CEq (Cell equivalents) (A) and RNA /1μg of total RNA (B) from vaginal biopsies at the indicated times after infection were quantified by *gag*-qPCR (normalized on albumin content) and by RT-qPCR (normalized on RNA content), respectively. The dotted line indicates the lower limit of detection (LLOD) of the assay. Bars represent median ± IQR.
(PDF)

S7 Fig SIV DNA loads in different tissues at necropsy. Viral DNA loads in each tissue were measured by SIV *gag*-qPCR and normalized on albumin copies. Copies of SIV DNA/ 10^4^ CEq (Cell equivalents) for JEJ (jejunal) and indicated tissues are shown in the upper row. The lower row shows copies of SIV DNA/ 10^4^ CEq (Cell equivalents) in lymph nodes (AX = axillary, ING = inguinal, MLN = mesenteric lymph nodes). The dotted line indicates the lower limit of detection (LLOD) of the assay. Bars represent median ± IQR.
(PDF)

S8 Fig Gating strategy for IL-17A and IFN-γ producing cells shown in Fig 3. (A-D) Gating strategy for IL-17A producing cells in PBMC at the acute time point. Cells were gated on lymphocytes, singlets and live (Aqua negative). A) T cells gating in PMA/Ionomycin stimulated PBMC (CD4^+^ T cells upper row; CD8^+^ T cells lower row) B) corresponding unstimulated sample (CD4^+^ T cells upper row; CD8^+^ T cells lower row); C-D) NK cells gating in PMA/Ionomycin stimulated (C) or unstimulated (D) PBMC. (E-H) Gating strategy for IL-17A producing cells in colorectal biopsies at the acute time point. Cells were gated on lymphocytes, singlets and live (Aqua negative). E) T cells gating in PMA/Ionomycin stimulated mononuclear cells isolated from colorectal biopsies (CD4^+^ T cells upper row; CD8^+^ T cells lower row) B) corresponding unstimulated sample (CD4^+^ T cells upper row; CD8^+^ T cells lower row); C-D) NK cells gating in PMA/Ionomycin stimulated (C) or unstimulated (D) mononuclear cells isolated from colorectal biopsies (E-H) Gating strategy for IFN-γ producing cells in colorectal tissue at necropsy (and baseline). I) PMA/Ionomycin stimulated J) unstimulated sample.
(PDF)

S9 Fig No difference in IL-17 and IFN-γ producing cells in the blood and gut of macaques before treatment. (A-B) The frequency of IL-17-secreting cells within the indicated subsets in blood (A) and colorectal biopsies (B) collected 2 weeks before treatment are shown. (C) The frequency of IFN-γ-secreting cells within CD8^+^ T cells in colorectal biopsies 2 weeks before treatment are shown. (A-C) NK-like cells were defined as CD3^-^NKG2A^+^ in the blood and CD3^-^NKp44^+^ in the colorectal tissue. Bars represent median ± IQR. Data from the treatment groups were compared with the control by Kruskal-Wallis test and the results of the Dunn’s multiple comparisons post-hoc test and the Mann-Whitney test to compare the treatment groups between each other are shown (*p*-value of * α<0.05 and ** α<0.01 were considered significant).
(PDF)

S10 Fig Rh-α_4_β_7_-VRC01-treated macaques have higher levels of circulating CCR6^+^ CD4^+^ T cells in the chronic phase. Around week 20 p.i., blood T cells were phenotyped by flow cytometry. The frequency of the subsets (the frequency of CCR6^+^ CD95^-^ within CD4^+^ T cells) that significantly differed among the treatment group is shown. The results of the Dunn’s multiple comparisons post-hoc test (after the Kruskal-Wallis test controlled for multiple comparisons) and the Mann-Whitney test to compare the treatment groups between each other are shown (*p*-value of * α<0.05, was considered significant). Bars represent median ± IQR.
(PDF)

S11 Fig Blood T cell responses against the consensus B envelope peptide pool. A-B) PBMCs isolated around 18 weeks post infection were stimulated with pooled 15-mer peptides with an 11aa overlap from the consensus B envelope protein for 5 hours. The frequency of cells secreting the indicated cytokines is shown for the CD4^+^ (A) and CD8^+^ (B) T cell subsets after subtraction of the baseline values (in absence of peptides). The results of the Dunn’s multiple comparisons post-hoc test (after the Kruskal-Wallis test controlled for multiple comparisons) and the Mann-Whitney test to compare the treatment groups between each other are shown (*p*-value of * α<0.05, ** α<0.01 and *** α<0.001 were considered significant). Bars represent median ± IQR.
(PDF)

S12 Fig Alignment of the V1-V2 region of the consensus B and SHIV_AD8-EO_ envelope sequences. Peptides of 20aa (overlapping 14aa) spanning the region shown in yellow were synthetized and used to probe T cell and antibody responses.
(PDF)

S13 Fig CD8^+^ T cell responses to the V1V2 peptides correlated with viral load. The frequencies of CD8^+^ T cells producing TNF-α in response to V1V2 peptides in the blood of VRC01-Rh-α_4_β_7_ and VRC01 treated macaques (shown in Fig 5B) are plotted against the viral loads. Control macaques had undetectable responses. Linear regression p value and R-square are shown for the VRC01-Rh-α_4_β_7_ group (Spearman non-parametric correlation p = 0.04 and r = -0.69). No significant correlation was found for the VRC01 group.
(PDF)

S14 Fig Peptide scan of anti-envelope antibodies. Sera from 4 animals with the highest anti-envelope antibodies in each treatment group were analyzed by peptide scan against consensus B envelope peptides. 7 SHIV-AD8-specific peptides replaced the corresponding peptides in the V1-V2 loop region.
(PDF)

S15 Fig Panels used for flow cytometry analysis of cell subset and T cell responses. Analyses during the acute phase were done with samples collected at week 3 or 4 post-infection, while chronic samples were collected from week 18 to 22 post-infection. The tables list the mAbs used for the flow cytometry analysis.
(PDF)
