# Supplementary figures and images for: Delayed vaginal SHIV infection in VRC01 and anti-α4β7 treated rhesus macaques
Source: PLoS Pathog. 2019 May 13;15(5):e1007776. doi: 10.1371/journal.ppat.1007776 (PMC6533011; doi:10.1371/journal.ppat.1007776)

# Figure S1

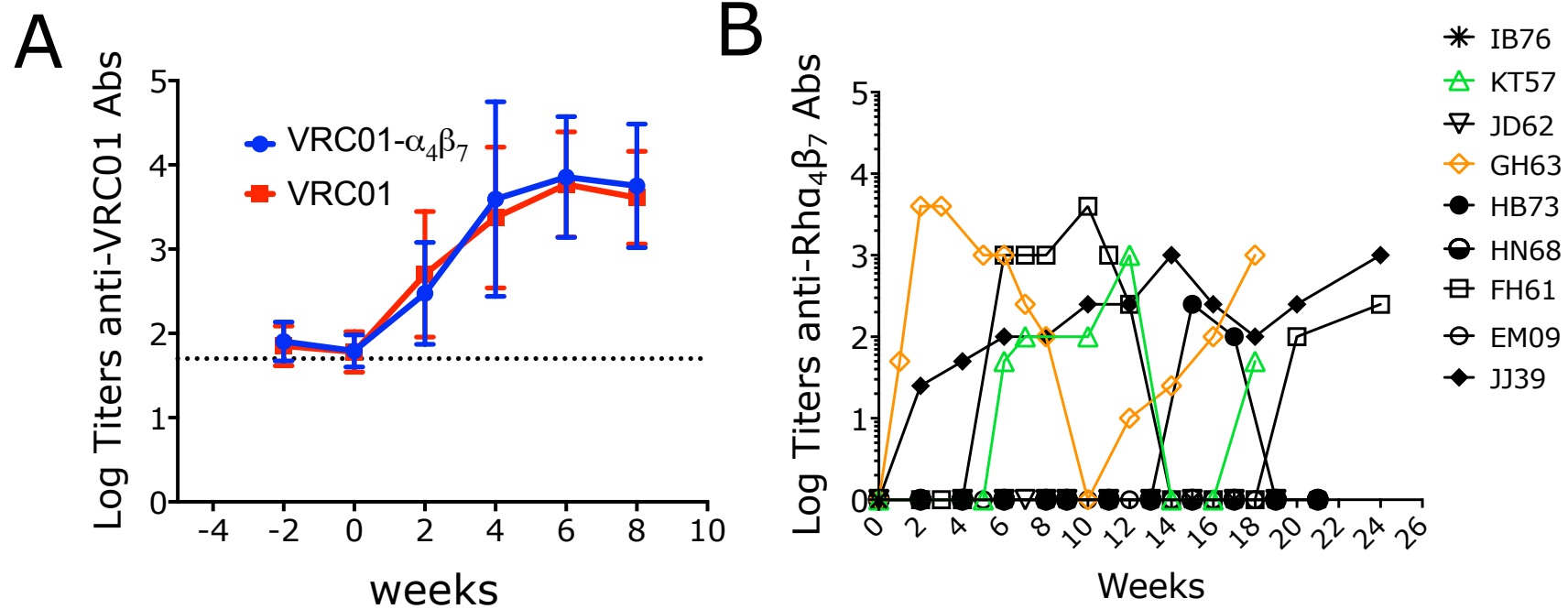

Supplement: S1 Fig — Mean ± SEM of the endpoint titers of anti-VRC01 antibodies in the VRC01-alone and VRC01-α4β7 group are shown at baseline and for the first 8 weeks past-infusion. B) Endpoint titers of anti-Rh-α4β7 antibodies are shown for the VRC01-α4β7 group from baseline to the necropsy. (PDF) [file ppat.1007776.s001.pdf]

Figure S2

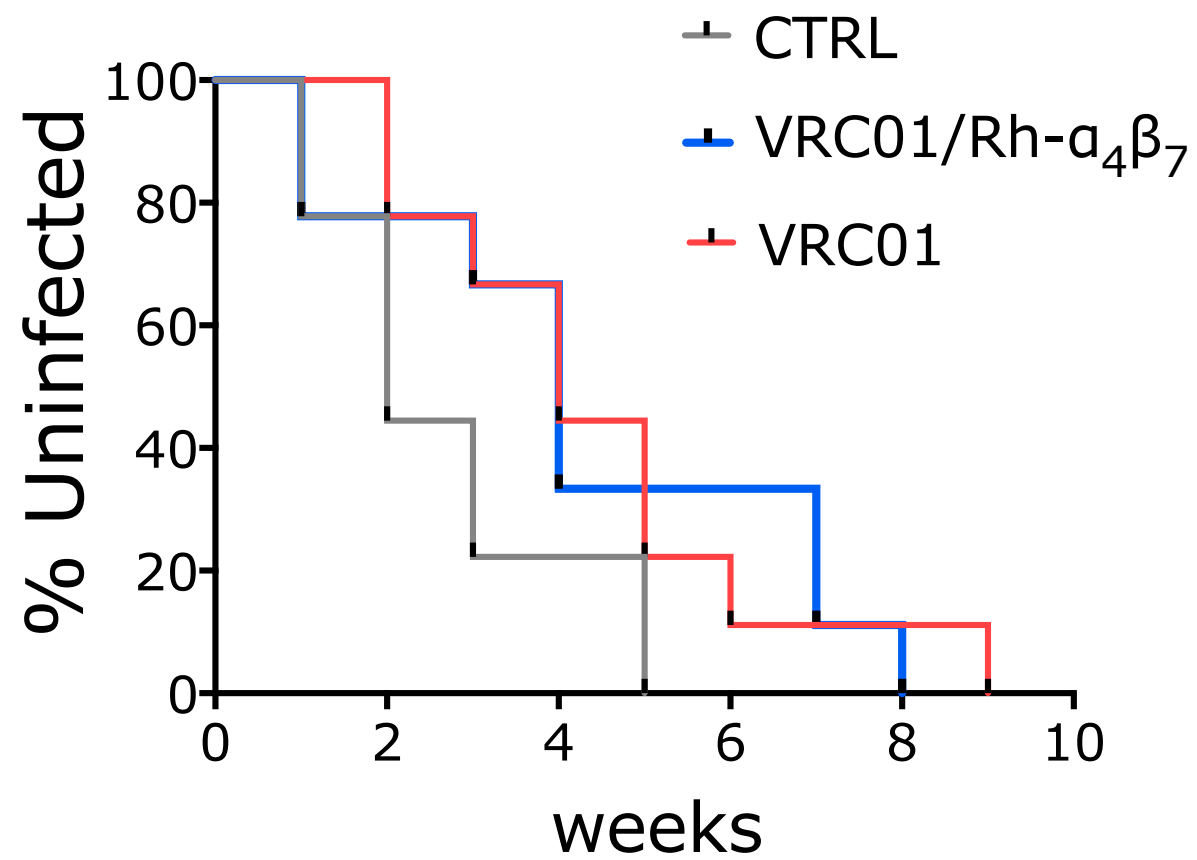

Supplement: S2 Fig — Kaplan-Meier curves generated with time to first viral detection in plasma are shown. Curves were compared with the Log-rank test and Gehan-Breslow-Wilcoxon test and no comparison was significant after Bonferroni correction for multiple comparisons. (PDF) [file ppat.1007776.s002.pdf]

Figure S5

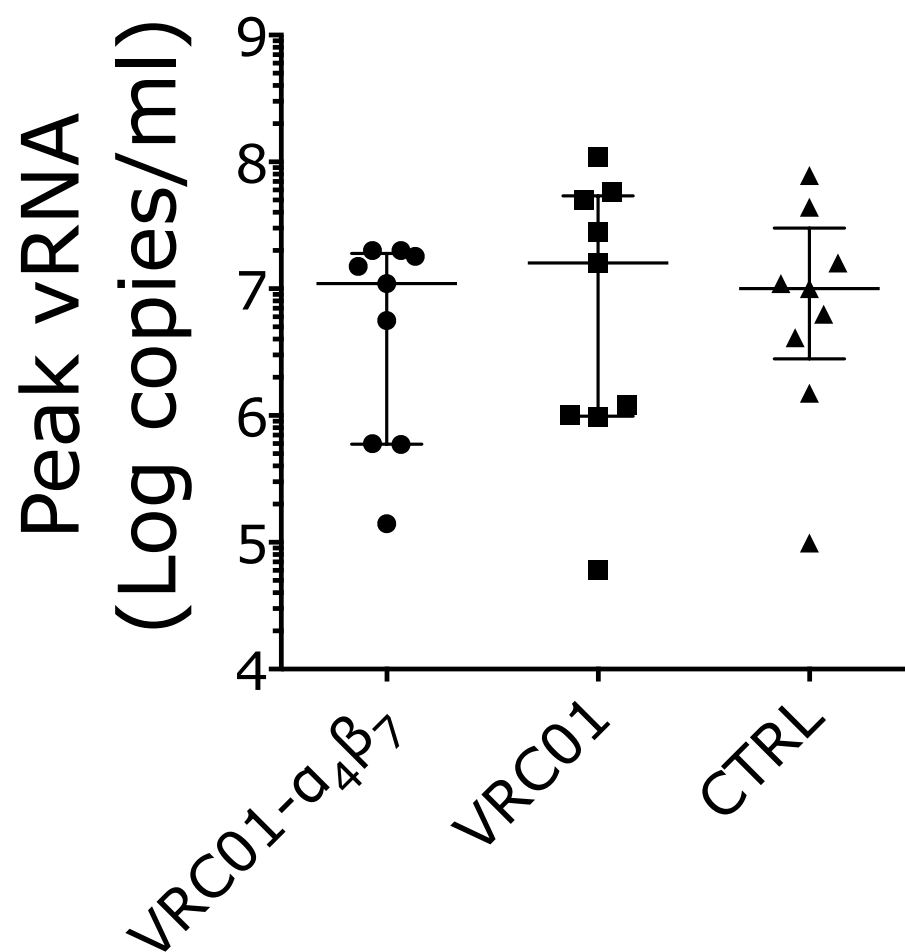

Supplement: S5 Fig — Highest level of SIV RNA copies in plasma reached within the first 5 weeks of infection in each animal is shown. Bars represent median ± IQR. (PDF) [file ppat.1007776.s005.pdf]

Figure S6

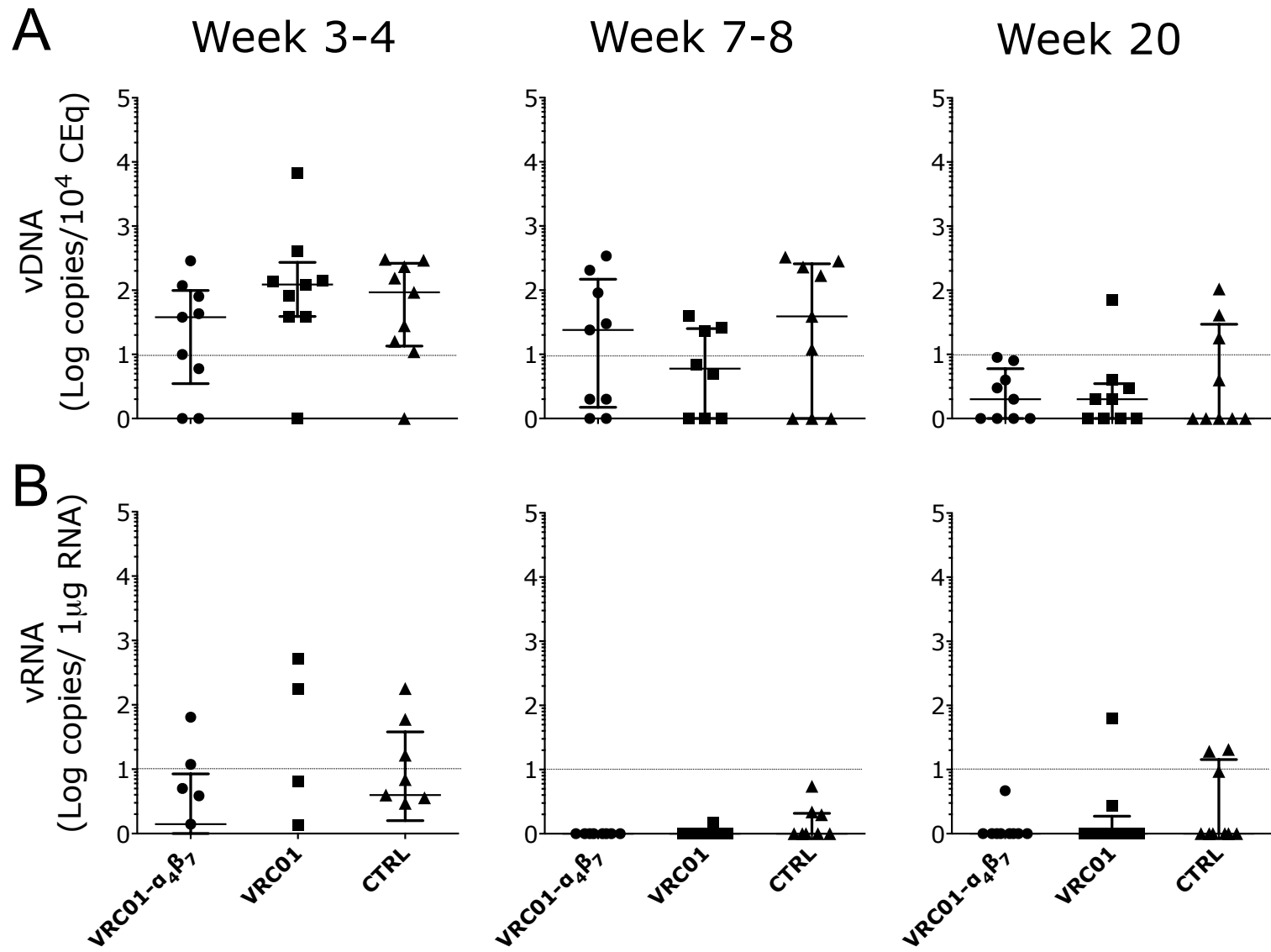

Supplement: S6 Fig — Copies of SIV DNA/ 104 CEq (Cell equivalents) (A) and RNA /1μg of total RNA (B) from vaginal biopsies at the indicated times after infection were quantified by gag-qPCR (normalized on albumin content) and by RT-qPCR (normalized on RNA content), respectively. The dotted line indicates the lower limit of detection (LLOD) of the assay. Bars represent median ± IQR. (PDF) [file ppat.1007776.s006.pdf]

Figure S7

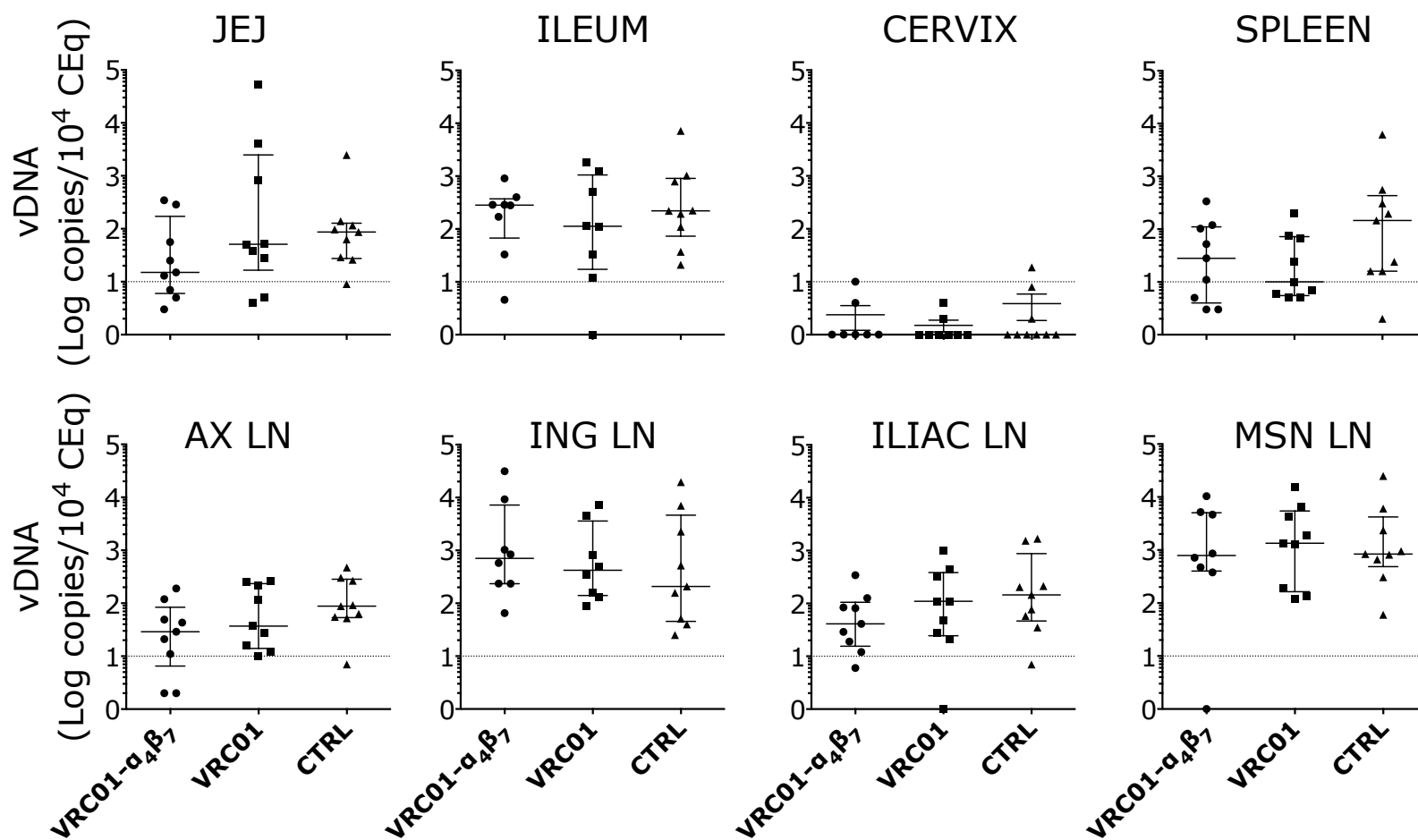

Supplement: S7 Fig — Viral DNA loads in each tissue were measured by SIV gag-qPCR and normalized on albumin copies. Copies of SIV DNA/ 104 CEq (Cell equivalents) for JEJ (jejunal) and indicated tissues are shown in the upper row. The lower row shows copies of SIV DNA/ 104 CEq (Cell equivalents) in lymph nodes (AX = axillary, ING = inguinal, MLN = mesenteric lymph nodes). The dotted line indicates the lower limit of detection (LLOD) of the assay. Bars represent median ± IQR. (PDF) [file ppat.1007776.s007.pdf]

Figure S8

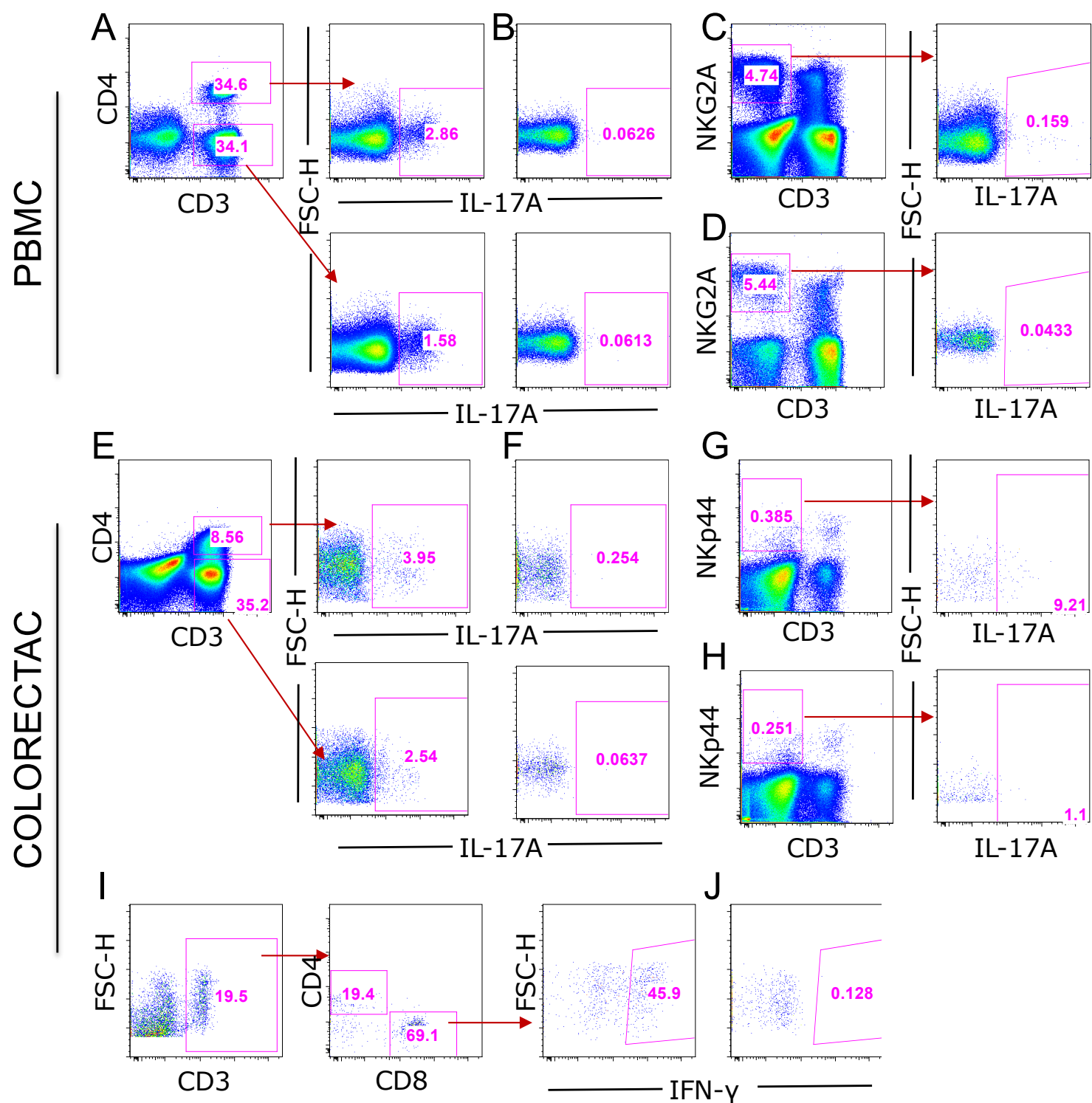

Supplement: S8 Fig — (A-D) Gating strategy for IL-17A producing cells in PBMC at the acute time point. Cells were gated on lymphocytes, singlets and live (Aqua negative). A) T cells gating in PMA/Ionomycin stimulated PBMC (CD4+ T cells upper row; CD8+ T cells lower row) B) corresponding unstimulated sample (CD4+ T cells upper row; CD8+ T cells lower row); C-D) NK cells gating in PMA/Ionomycin stimulated (C) or unstimulated (D) PBMC. (E-H) Gating strategy for IL-17A producing cells in colorectal biopsies at the acute time point. Cells were gated on lymphocytes, singlets and live (Aqua negative). E) T cells gating in PMA/Ionomycin stimulated mononuclear cells isolated from colorectal biopsies (CD4+ T cells upper row; CD8+ T cells lower row) B) corresponding unstimulated sample (CD4+ T cells upper row; CD8+ T cells lower row); C-D) NK cells gating in PMA/Ionomycin stimulated (C) or unstimulated (D) mononuclear cells isolated from colorectal biopsies (E-H) Gating strategy for IFN-γ producing cells in colorectal tissue at necropsy (and baseline). I) PMA/Ionomycin stimulated J) unstimulated sample. (PDF) [file ppat.1007776.s008.pdf]

Figure S9

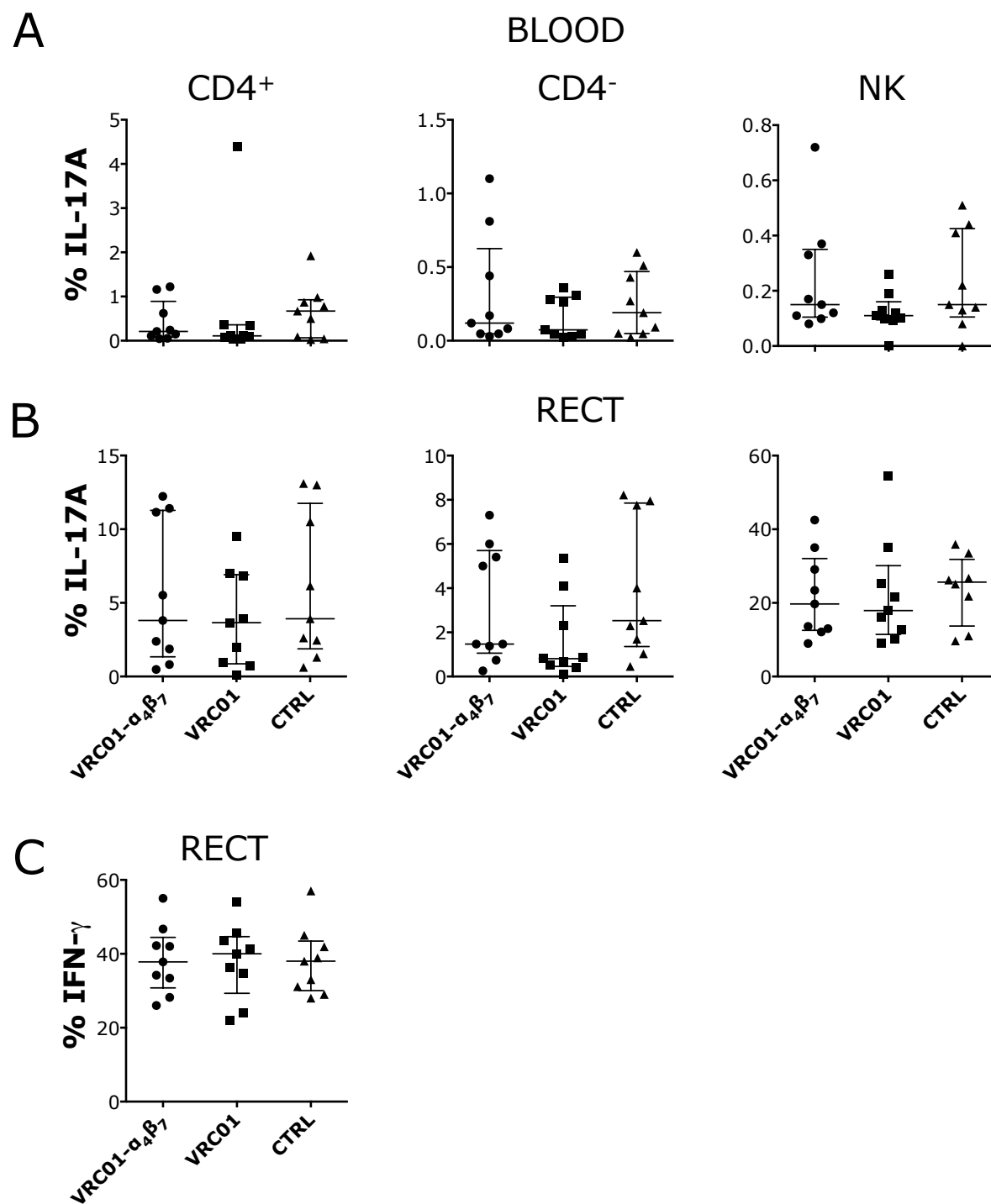

Supplement: S9 Fig — (A-B) The frequency of IL-17-secreting cells within the indicated subsets in blood (A) and colorectal biopsies (B) collected 2 weeks before treatment are shown. (C) The frequency of IFN-γ-secreting cells within CD8+ T cells in colorectal biopsies 2 weeks before treatment are shown. (A-C) NK-like cells were defined as CD3-NKG2A+ in the blood and CD3-NKp44+ in the colorectal tissue. Bars represent median ± IQR. Data from the treatment groups were compared with the control by Kruskal-Wallis test and the results of the Dunn’s multiple comparisons post-hoc test and the Mann-Whitney test to compare the treatment groups between each other are shown (p-value of * α<0.05 and ** α<0.01 were considered significant). (PDF) [file ppat.1007776.s009.pdf]

Figure S10

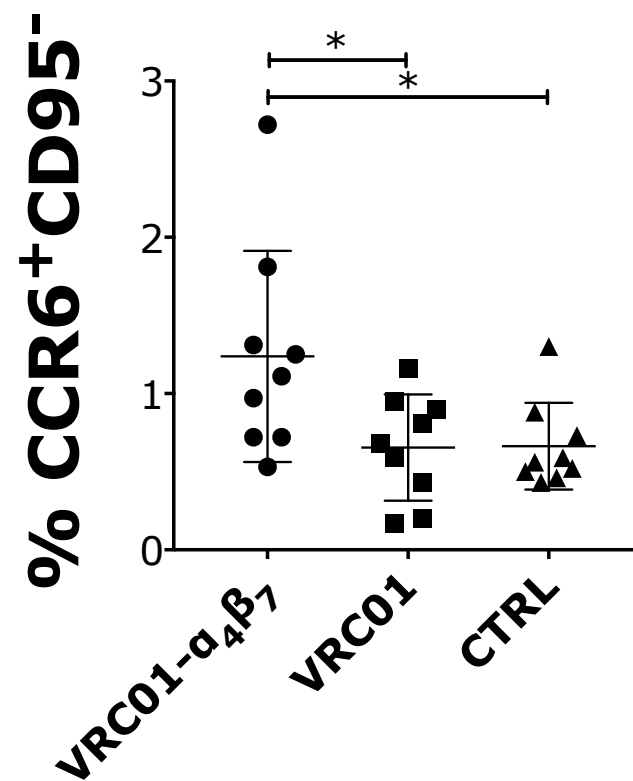

Supplement: S10 Fig — Around week 20 p.i., blood T cells were phenotyped by flow cytometry. The frequency of the subsets (the frequency of CCR6+ CD95- within CD4+ T cells) that significantly differed among the treatment group is shown. The results of the Dunn’s multiple comparisons post-hoc test (after the Kruskal-Wallis test controlled for multiple comparisons) and the Mann-Whitney test to compare the treatment groups between each other are shown (p-value of * α<0.05, was considered significant). Bars represent median ± IQR. (PDF) [file ppat.1007776.s010.pdf]

Figure S11

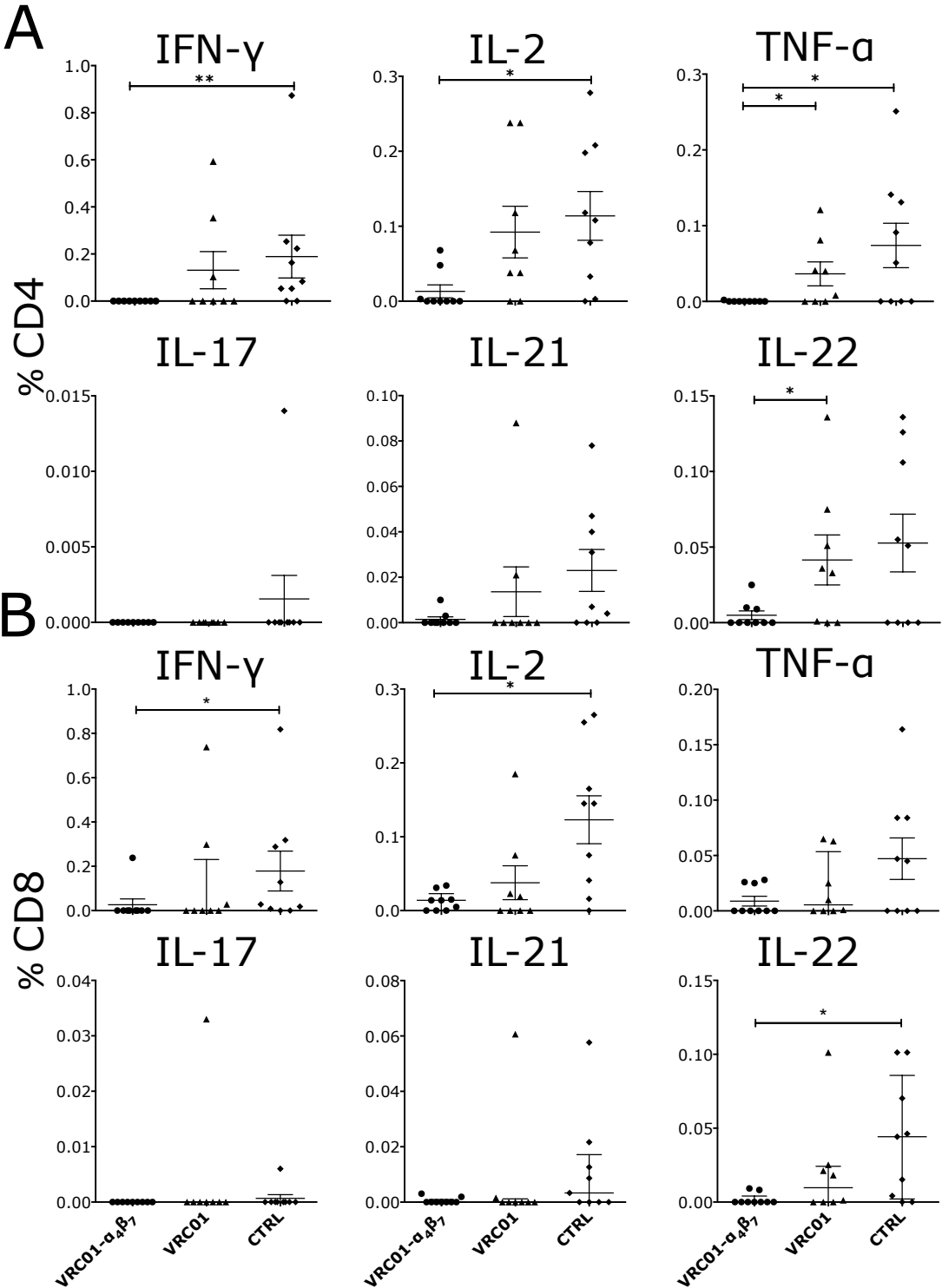

Supplement: S11 Fig — A-B) PBMCs isolated around 18 weeks post infection were stimulated with pooled 15-mer peptides with an 11aa overlap from the consensus B envelope protein for 5 hours. The frequency of cells secreting the indicated cytokines is shown for the CD4+ (A) and CD8+ (B) T cell subsets after subtraction of the baseline values (in absence of peptides). The results of the Dunn’s multiple comparisons post-hoc test (after the Kruskal-Wallis test controlled for multiple comparisons) and the Mann-Whitney test to compare the treatment groups between each other are shown (p-value of * α<0.05, ** α<0.01 and *** α<0.001 were considered significant). Bars represent median ± IQR. (PDF) [file ppat.1007776.s011.pdf]

Figure S13

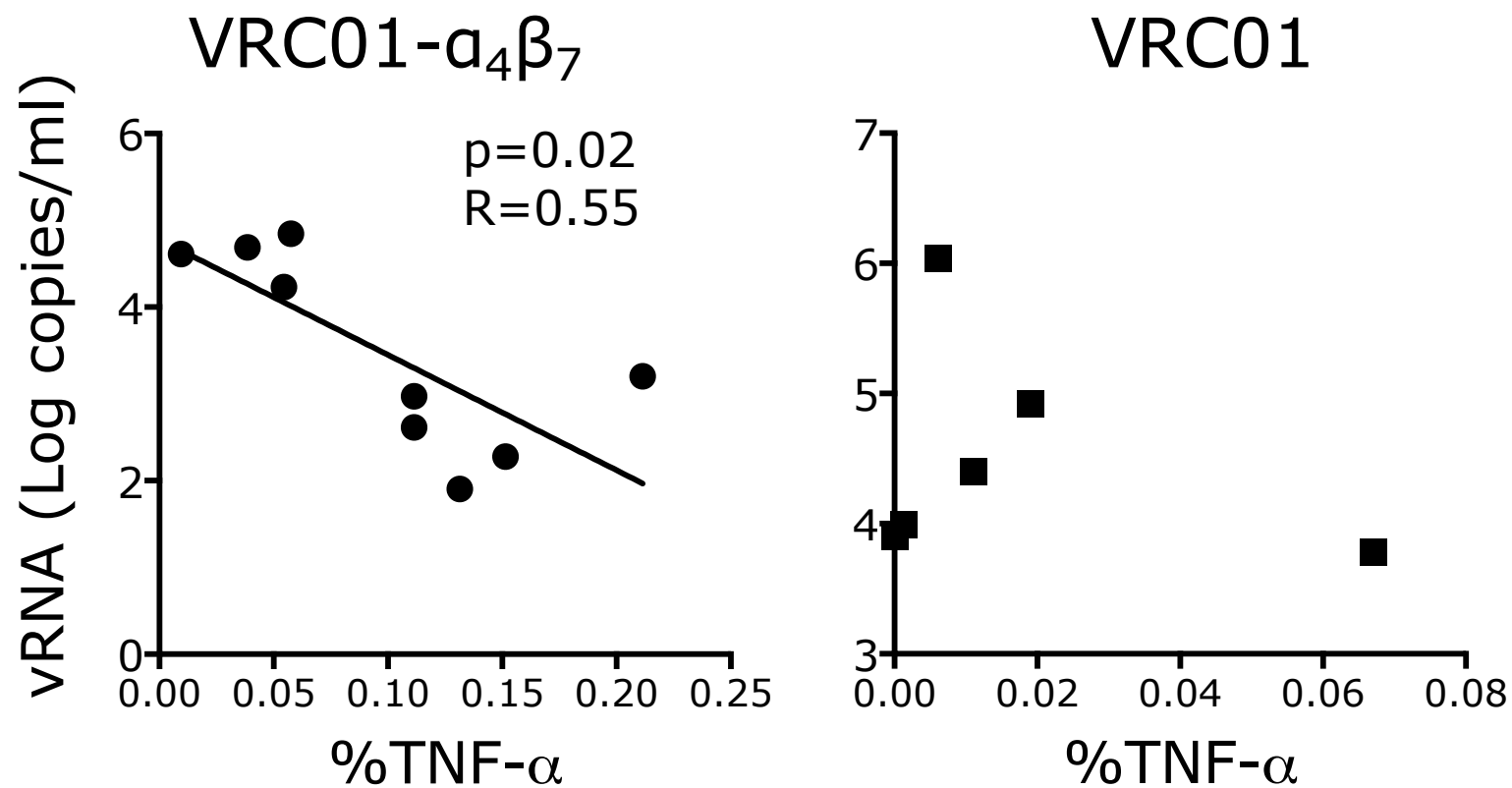

Supplement: S13 Fig — The frequencies of CD8+ T cells producing TNF-α in response to V1V2 peptides in the blood of VRC01-Rh-α4β7 and VRC01 treated macaques (shown in Fig 5B) are plotted against the viral loads. Control macaques had undetectable responses. Linear regression p value and R-square are shown for the VRC01-Rh-α4β7 group (Spearman non-parametric correlation p = 0.04 and r = -0.69). No significant correlation was found for the VRC01 group. (PDF) [file ppat.1007776.s013.pdf]

Figure S14

Rh- $\alpha 4\beta 7$  + VRC01

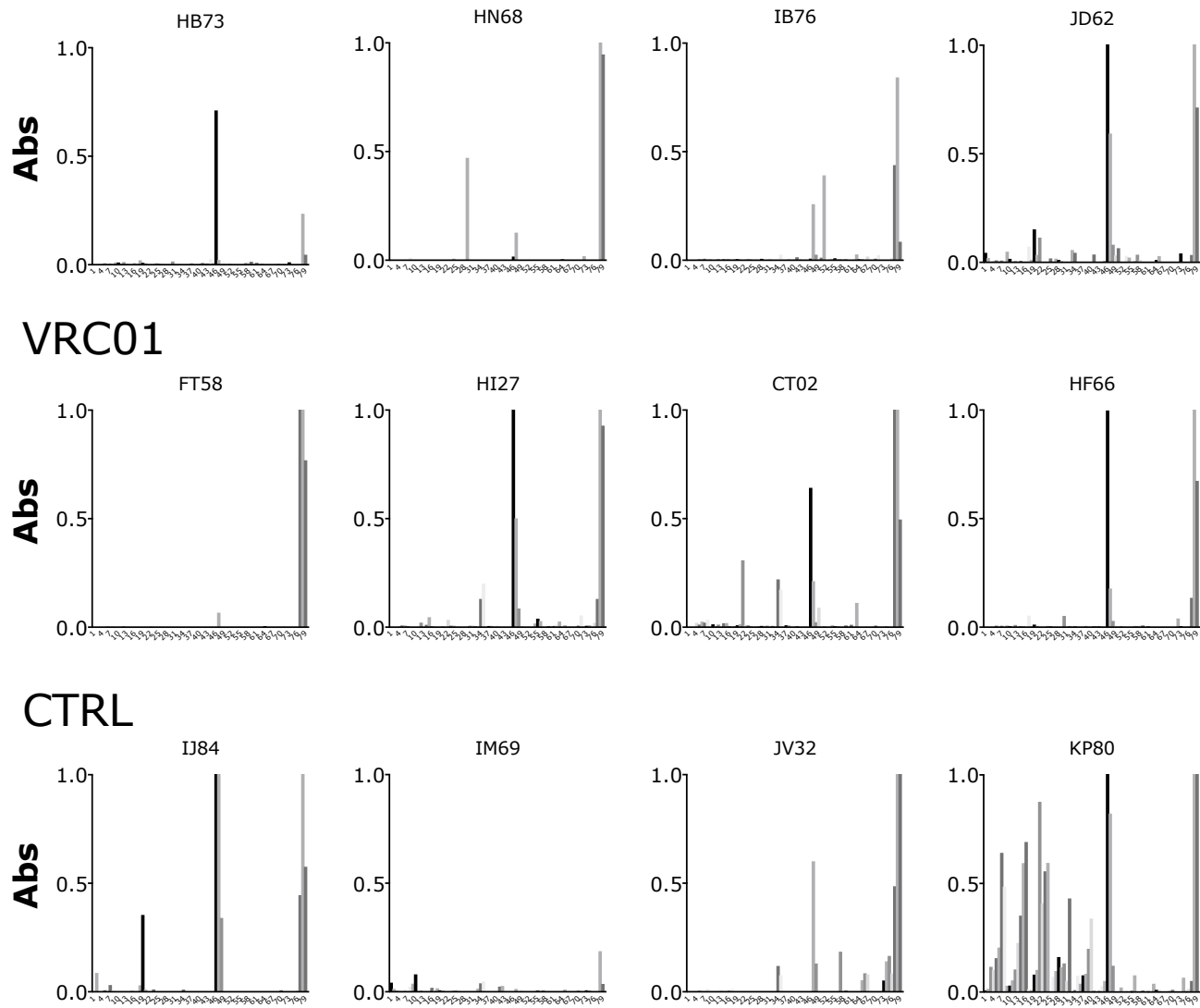

Supplement: S14 Fig — Sera from 4 animals with the highest anti-envelope antibodies in each treatment group were analyzed by peptide scan against consensus B envelope peptides. 7 SHIV-AD8-specific peptides replaced the corresponding peptides in the V1-V2 loop region. (PDF) [file ppat.1007776.s014.pdf]
