# Supplementary material for: Delayed vaginal SHIV infection in VRC01 and anti-α4β7 treated rhesus macaques
Source: PLoS Pathog. 2019 May 13;15(5):e1007776. doi: 10.1371/journal.ppat.1007776 (PMC6533011; doi:10.1371/journal.ppat.1007776)
Supplement: S3 Fig — RNA was isolated from PBMC of each animal and cDNA prepared. Gene-specific PCRs were run, and the product sequenced. Animals are listed in order of treatment with the first 9 animals belonging to the VRC01 + Rh-α4β7 group, then the 9 animals from the VRC01-only group and finally the 9 animals in the control group. The alleles in yellow had 2 different nucleotides present at more than a single SNP. They were inferred based on the allele frequency in the population. In bold are the 2 animals with very low VRC01 concentrations. (PDF) [file ppat.1007776.s003.pdf]

Figure S3

	CD16				CD64			
**RM**	**3A-1**	**3A-2**	**3A-3**		**Allele 1**	**Allele 2**	**Allele 6**	**Allele 7**
IB76		XX				X	X	
**KT57**	X	X			XX			
JD62	XX				XX			
**GH63**		XX			XX			
HB73	X	X			X		X	
HN68	XX				XX			
FH61		XX			XX			
EM09	X	X			XX			
JJ39	X	X			X		X	
HF66		XX			XX			
KT03	X	X			XX			
FT58	X	X			XX			
HI27	XX				X		X	
IK06	X	X			X			X
EN78		XX			X	X		
ED86	X	X			X	X		
CT02	X	X			X		X	
CJ36		X	X		XX			
HR40	X	X					X	X
KP80	XX				X			X
JV32	X	X				X		X
IV98	X	X			XX			
IM69	X	X			XX			
IJ84	XX				XX			
IK53	X	X			XX			
HN46		XX			XX			
HM14	X	X			XX			

**CD64 Alleles**				
Position a.a	46	78	85	154
Mature peptide aa	41 (nt)	73 (nt)	80 (nt)	149 (nt)
allele				
1	V (a)	V (a)	R (c)	L (g)
2	V (a)	A (g)	R (c)	L (g)
3	V (a)	V (a)	S (g)	L (g)
4	V (a)	V (a)	S (g)	F (a)
5	V (a)	V (a)	R (c)	F (a)
6	A (g)	V (a)	R (c)	L (g)
7	A (g)	V (a)	S (g)	L (g)
8	V (a)	V (a)	S (g)	L (g)
9	A (g)	V (a)	S (g)	F (a)
