# Supplementary material for: Delayed vaginal SHIV infection in VRC01 and anti-α4β7 treated rhesus macaques
Source: PLoS Pathog. 2019 May 13;15(5):e1007776. doi: 10.1371/journal.ppat.1007776 (PMC6533011; doi:10.1371/journal.ppat.1007776)
Supplement: S4 Fig — RNA was isolated from PBMC of each animal and cDNA prepared. Gene-specific PCRs were run and the product sequenced. Animals are listed in order of treatment with the first 9 animals belonging to the VRC01 + Rh-α4β7 group, then the 9 animals from the VRC01-only group and finally the 9 animals in the control group. In green are highlighted the animals with the most common allotype. In bold are the 2 animals with very low VRC01 concentrations. (PDF) [file ppat.1007776.s004.pdf]

Figure S4

a.a.	R/M	V/I	T/P	A/T	K/I	A/S	N/K	H/P	D/N	Q/R	A/V	I/V	+D
**nt position**	**272**	**343**	**385**	**463**	**482**	**484**	**492**	**500**	**505**	**527**	**686**	**691**	**between897/898**
**nt**	**G/T**	**G/A**	**A/C**	**G/A**	**A/T**	**G/T**	**C/A**	**A/C**	**G/A**	**A/G**	**C/T**	**a/g**	**GAT added**
**animal Code**													
IB76	T	G	A	G	A	T	C	A	A	A	T	A	NO
**KT57**	G/T	G	A	G	A	T	C	A	A	A	T/C	A	GAT/NO
JD62	G/T	G	A/C	A	T	G/T	A	A	G	A	C	G/A	NO
**GH63**	T	G	A/C	G/A	A/T	T	A	A	A/G	A	T/C	A	NO
HB73	T	G	A	G	A	T	C	A	A	A	T	A	NO
HN68	T	G	A	G	A	T	C	A	A	A	T	A	NO
FH61	T	G	A	G	A	T	C	A	A	A	T/C	A	NO
EM09	T	G	A/C	G/A	A/T	T	C	A	A/G	A	T/C	G/A	NO
JJ39	G/T	G	A/C	G/A	A/T	T	C/A	A	A/G	A	T/C	G/A	NO
HF66	T	G	A	G	A	T	C	A	A	A	T	A	NO
KT03	G/T	G	A	G	A	T	C	A	A	A	T/C	A	GAT/NO
FT58	T	G	A	G	A	T	C	A	A	A	T	A	NO
HI27	T	G	A	G	A	T	C	A	A	A	T	A	NO
IK06	T	G	A	G	A	T	C	A	A	A	T	A	NO
EN78	T	G	A	G	A	T	C	A	A	A	T	A	NO
ED86	T	G	A	G	A	T	C	A	A	A	T	A	NO
CT02	T	G	A	G	A	T	C	A	A	A	T	A	NO
CJ36	G/T	G	A/C	G/A	A/T	T	C/A	A	A/G	A	T/C	G/A	GAT/NO
HR40	T	G	A	G	A	T	C	A	A	A	T	A	NO
KP80	T	G	A	G	A	T	C	A	A	A	T	A	NO
JV32	T	G	A	G	A	T	C	A	A	A	T	A	NO
IV98	T	G	A	G	A	T	C	A	A	A	T	A	NO
IM69	T	G	A	G	A	T	C	A	A	A	T	A	NO
IJ84	T	G	A	G	A	T	C	A	A	A	T	A	NO
IK53	T	G	A	G	A	T	C	A	A	A	T	A	NO
HN46	G/T	G	A/C	G/A	A/T	T	C/A	A/C	A/G	A/G	T/C	G/A	GAT/NO
HM14	T	G	A/C	G	A/T	T	C	A	A/G	A/G	T/C	G/A	GAT/NO
