# Supplementary material for: Delayed vaginal SHIV infection in VRC01 and anti-α4β7 treated rhesus macaques
Source: PLoS Pathog. 2019 May 13;15(5):e1007776. doi: 10.1371/journal.ppat.1007776 (PMC6533011; doi:10.1371/journal.ppat.1007776)
Supplement: S12 Fig — Peptides of 20aa (overlapping 14aa) spanning the region shown in yellow were synthetized and used to probe T cell and antibody responses. (PDF) [file ppat.1007776.s012.pdf]

Figure S12

```
Majority                      QEVVLENVTENFNMWKNNMVEQMHEDIISLWDQSLKPCVKLTPLCVTLNCTDXXNXTNXXXXSSXEXMXXGEIKNCSFNI
                                       90        100       110       120       130       140       150       160
Consensus B Env protein.pro   QEVVLENVTENFNMWKNNMVEQMHEDIISLWDQSLKPCVKLTPLCVTLNCTDLMNATNTTNSSSGEKMEKGEIKNCSFNI  160
SHIV_AD8 Env.pro              ....................................................WG.V..I--N..S.E.-R..........  157

Majority                      TTSIRDKVXXXYALFYXLDVVPIDNDNTSXYRLISCNTSXITQACPKVSFEPIPIHYCXPAGFAILKCXDKKFNGTGPCX
                                       170       180       190       200       210       220       230       240
Consensus B Env protein.pro   TTSIRDKVQKEYALFYKLDVVPIDNDNTSSYRLISCNTSVITQACPKVSFEPIPIHYCAPAGFAILKCNDKKFNGTGPCT  240
SHIV_AD8 Env.pro              ........KED.....R............-.........T..................T.........K..........K  236
```
