# Supplementary material for: Delayed vaginal SHIV infection in VRC01 and anti-α4β7 treated rhesus macaques
Source: PLoS Pathog. 2019 May 13;15(5):e1007776. doi: 10.1371/journal.ppat.1007776 (PMC6533011; doi:10.1371/journal.ppat.1007776)
Supplement: S15 Fig — Analyses during the acute phase were done with samples collected at week 3 or 4 post-infection, while chronic samples were collected from week 18 to 22 post-infection. The tables list the mAbs used for the flow cytometry analysis. (PDF) [file ppat.1007776.s015.pdf]

Figure S15

PBMC acute	Color	Clone	Manufacturer
CD3	V450	SP34-2	BD Biosciences
CD4	BUV395	L200	BD Biosciences
CD95	PercP-Fluor710	DX2	eBioscience
CCR6	PE-Dazzle 594	G034E3	BioLegend
NKG2A	PE	REA110	Miltenyi
alpha4	AF700	7.2R	Novus Biologicals
CXCR3	AF488	G025H7	BioLegend
IL-17A	PE-Cy7	Ebio64DEC17	eBioscience
P27	AF647H	2F12	NIH AIDS Reagent P
CCR7	BV605	G043H7	BioLegend
IFN-γ	APC-efluor780	4S.B3	eBioscience

PBMC stimulation	Color	Clone	Manufacturer
CD3	V450	SP34-2	BD Biosciences
CD8	PE-CF594	RPA-T8	BD Biosciences
CD4	BUV395	L200	BD Biosciences
NKG2A	PE Vio 770	REA110	Miltenyi
IL-17A	APC-eFluor 780	Ebio64DEC17	eBioscience
IFN-γ	AF700	B27	BD Biosciences
IL-2	Brilliant Violet 605	MQ1-17H12	BioLegend
IL-21	APC	3A3-N2	BioLegend
IL-22	PercP-Fluor710	IL22JOP	eBioscience
TNF-α	FITC	MAb11	BioLegend

PBMC (chronic)	Color	Clone	Manufacturer
CD3	Alexa Fluor 700	SP34-2	BD Biosciences
CD4	BUV395	L200	BD Biosciences
CXCR5	FITC	710D82.1	NHP Reagent R
CXCR3	PercP-Cy5.5	G025H7	BD Biosciences
CD95	V450	DX2	BD Biosciences
CD127	PE-Cy7	eBioRDR5	eBioscience
CD25	APC-eFluor 780	BC96	BioLegend
CCR6	PE-Dazzle 594	G034E3	BioLegend
CD103	PE	B-Ly7	eBioscience
CD69	Brilliant Violet 605	FN50	BD Biosciences
p27	APC	2F12	NIH AIDS Reagent P.

Rectal acute	Color	Clone	Manufacturer
CD3	V450	SP34-2	BD Biosciences
CD4	BUV395	L200	BD Biosciences
Nkp44	PercP-Cy5.5	P44	BioLegend
CCR6	PE-Dazzle 594	G034E3	BioLegend
α4	AF700	7.2R	Novus Biologicals
CXCR3	AF488	G025H7	BioLegend
IL-17A	PE-Cy7	Ebio64DEC17	eBioscience
NKG2A	PE	REA110	Miltenyi
CD20	AF700	2H7	BD Biosciences
IgA	In house APC-Cy7	10F12	NHP Reagent R

Tissue stimulation	Color	Clone	Manufacturer
CD3	V450	SP34-2	BD Biosciences
CD8	PE-CF594	RPA-T8	BD Biosciences
CD4	BUV395	L200	BD Biosciences
Nkp44 (CD336)	PercP-Cy5.5	P44-8	BioLegend
CCR6 (DcR2)	PE-Cy7	G034E3	BioLegend
TNF-α	FITC	MAb11	BioLegend
IL-17A	APC-eFluor 780	Ebio64DEC17	eBioscience
IFN-γ	AF700	B27	BD Biosciences
IL-2	Brilliant Violet 605	MQ1-17H12	BioLegend
IL-21	APC	3A3-N2	BioLegend
p27	APC	2F12	NIH AIDS Reagent P

LN (chronic)	Color	Clone	Manufacturer
CD3	Alexa Fluor 700	SP34-2	BD Biosciences
CD4	BUV395	L200	BD Biosciences
CXCR5	FITC	710D82.1	NHP
CXCR3	PercP-Cy5.5	G025H7	BD Biosciences
CD95	V450	DX2	BD Biosciences
CD127	PE-Cy7	eBioRDR5	eBioscience
CD25	APC-eFluor 780	BC96	BioLegend
CCR6	PE-Dazzle 594	G034E3	BioLegend
CD103	PE	B-Ly7	eBioscience
CD69	Brilliant Violet 605	FN50	BD Biosciences
p27	APC	2F12	NIH AIDS Reagent P.
